# Anchoring Cu_1_ species over nanodiamond-graphene for semi-hydrogenation of acetylene

**DOI:** 10.1038/s41467-019-12460-7

**Published:** 2019-09-30

**Authors:** Fei Huang, Yuchen Deng, Yunlei Chen, Xiangbin Cai, Mi Peng, Zhimin Jia, Jinglin Xie, Dequan Xiao, Xiaodong Wen, Ning Wang, Zheng Jiang, Hongyang Liu, Ding Ma

**Affiliations:** 1 Shenyang National Laboratory for Materials Science, Institute of Metal Research, Chinese Academy of Sciences, 110016 Shenyang, P. R. China; 2 School of Materials Science and Engineering, University of Science and Technology of China, 230026 Hefei, P. R. China; 3 Beijing National Laboratory for Molecular Engineering, College of Chemistry and Molecular Engineering and College of Engineering, BIC-ESAT, Peking University, 100871 Beijing, P. R. China; 4 State Key Laboratory of Coal Conversion, Institute of Coal Chemistry, Chinese Academy of Sciences, 030001 Taiyuan, P. R. China; 5 University of Chinese Academy of Science, No. 19A Yuanquan Road, 100049 Beijing, P. R. China; 6 Department of Physics, Hong Kong University of Science and Technology, Clear Water Bay, Kowloon, Hong Kong SAR, P. R. China; 7 Center for Integrative Materials Discovery, Department of Chemistry and Chemical Engineering, University of New Haven, 300 Boston Post Road, West Haven, CT 06516 United States; 8 National Energy Center for Coal to Clean Fuel, Synfuels China Co., Ltd, Huairou District, 101400 Beijing, P. R. China; 9 Shanghai Institute of Applied Physics, Chinese Academy of Science, 201800 Shanghai, P. R. China; 10 Shanghai Synchrotron Radiation Facility, Zhangjiang Lad, Shanghai Advanced Research Institute, Chinese Academy of Science, 201210 Shanghai, P. R. China

**Keywords:** Catalysis, Materials chemistry

## Abstract

The design of cheap, non-toxic, and earth-abundant transition metal catalysts for selective hydrogenation of alkynes remains a challenge in both industry and academia. Here, we report a new atomically dispersed copper (Cu) catalyst supported on a defective nanodiamond-graphene (ND@G), which exhibits excellent catalytic performance for the selective conversion of acetylene to ethylene, i.e., with high conversion (95%), high selectivity (98%), and good stability (for more than 60 h). The unique structural feature of the Cu atoms anchored over graphene through Cu-C bonds ensures the effective activation of acetylene and easy desorption of ethylene, which is the key for the outstanding activity and selectivity of the catalyst.

## Abstract (web summary)

It is highly desired to explore cheap, non-toxic transition metals catalysts for semihydrogenation of acetylene. Here, isolated Cu atoms anchored onto a defective nanodiamond-graphene support exhibit robust catalytic performance in acetylene semihydrogenation in comparison with supported Cu clusters.

## Introduction

Selectively hydrogenating remnant acetylene in the raw olefin streams to ethylene while avoiding the over-hydrogenation to undesired ethane is a key industrial reaction to manufacture polymer-grade raw materials for the production of polyethylene^1–3^. The most commonly used industrial catalyst for the reaction is based on supported Pd nanoparticles (NPs) modified by Ag additives^4^. Although the Pd-Ag catalyst prevents the usage of toxic promoters such as lead or sulfur (Lindlar catalyst)^5^, the extremely high cost of Pd leaves ample room for improving the cost-effectiveness in catalyst design. In an effort to develop environment-friendly and cost-effective catalysts, various approaches have been pursued, including (i) reducing the amount of noble metals by “site-isolation” strategy or engineering a minimal ensemble^6–11^ and (ii) developing non-noble metals/metal oxides catalysts^12–18^.

The key to the first strategy is to prepare atomically dispersed metal catalysts, a burgeoning class of catalytic materials, in which isolated metal atoms were anchored on the solid supports^7,19–23^. Owing to their unique structural and electronic features, the atomically dispersed noble metal catalysts not only displayed unrivaled advantages for their maximal atomic utilization and high turnover frequency (TOF) but also strongly promoted the studies related to active site identifications and reaction mechanisms^23–28^. For the second approach, it is highly desired to develop new catalysts using cheap, non-toxic, and earth-abundant transition metals, such as Cu or Fe, to achieve comparable catalytic performance to that of Pd-based catalysts. Indeed, non-noble metal oxides have been investigated extensively for the development of low-cost and high-performance alkyne hydrogenation catalysts, including ceria^12–16^. Owing to limited H_2_ activation ability^29^, semihydrogenation of alkynes over these oxide catalysts normally required a relatively high-operating temperature. In an elegant work recently, Pardo et al. reported a metal–organic framework-based Fe(III)-O catalyst^18^. This single-site cationic species was active for acetylene hydrogenation at up to 150 °C, which is an important advance in non-noble metal catalyst for this reaction. Alternatively, earth-abundant metal especially Cu-based catalysts have been developed and evaluated for the reaction, suggesting that Cu, as an inexpensive and non-toxic catalyst, has an activity for acetylene hydrogenation over other aforementioned non-noble metals^30^. Yet, a small quantity of Pd promoter was still a must for achieving satisfactory catalytic performance^31,32^.

Herein we report the fabrication of cheap atomically dispersed Cu catalysts without other noble metals to effectively catalyze selective hydrogenation of acetylene. In the followings, we will first show adequate experimental evidences that isolated Cu atoms were anchored over the surface-defective nanodiamond–graphene (ND@G) support (Cu_1_/ND@G). Second, we will demonstrate that Cu_1_/ND@G possessed remarkable catalytic performance: high conversion (95%), high selectivity (98%), and good stability (for >60 h) for acetylene hydrogenation, compared to the Cu-cluster catalyst supported over the same host (denoted as Cu_n_/ND@G). Finally, by density functional theory (DFT) calculations, we will show that the unique structure of the atomically dispersed Cu catalyst facilitates the activation of acetylene and the desorption of ethylene, which is pivotal for the enhanced activity and selectivity of Cu_1_/ND@G compared to Cu_n_/ND@G.

## Results

### Synthesis and characterization of Cu_1_/ND@G and Cu_n_/ND@G

**Fig. 1:**
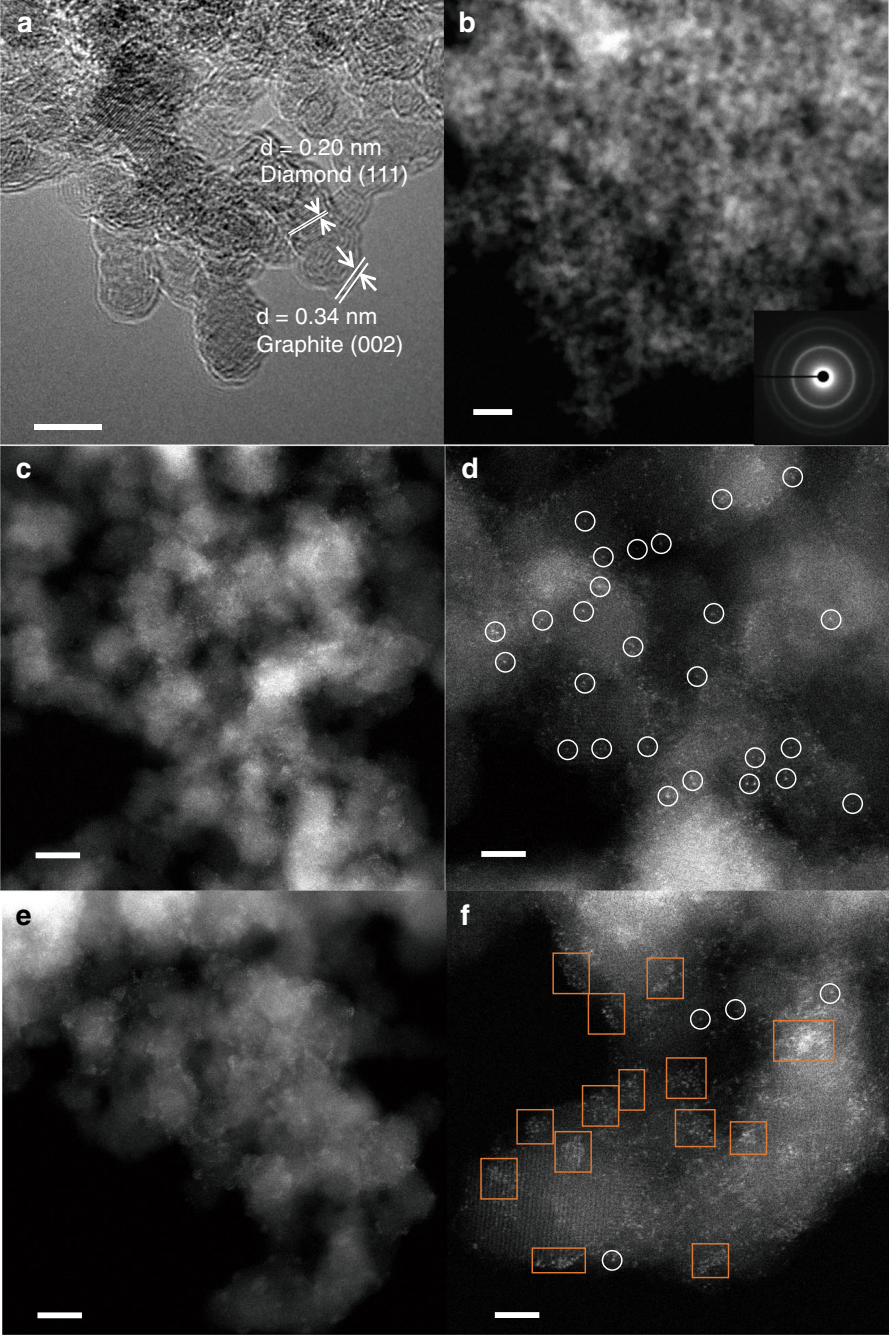
TEM characterization of ND@G support and Cu_1_/ND@G and Cu_n_/ND@G catalysts. **a** HRTEM image of ND@G nanocarbon support. Scale bar, 5 nm. **b** HAADF-STEM image of Cu_1_/ND@G at low magnification. Scale bar, 20 nm. **c** HAADF-STEM images of Cu_1_/ND@G at low magnification. Scale bar, 5 nm. **d** HAADF-STEM images of Cu_1_/ND@G at high magnification. Scale bar, 2 nm. **e** HAADF-STEM images of Cu_n_/ND@G at low magnification. Scale bar, 5 nm. **f** HAADF-STEM images of Cu_n_/ND@G at high magnification. Scale bar, 2 nm. (The inset attached to **b** is diamond’s diffraction rings’ image. Atomically dispersed Cu atoms are highlighted by white circles in **d** and Cu clusters are highlighted by orange squares in **f**.)

We prepared the Cu_1_/ND@G and Cu_n_/ND@G catalysts following the preparation procedure in the “Methods” section. Here we will probe the dispersion states of Cu atoms in these two different catalysts. The substrate ND@G features a thin graphene shell with abundant defects formed during the annealing of ND. High-resolution transmission electron microscope (HRTEM) images (see Fig. 1a and Supplementary Fig. 1), Raman spectra, and X-ray photoelectron spectroscopic (XPS) measurements (see Supplementary Figs. 3 and 4) revealed the unique defect-rich structure of ND@G. The highly defective few-layer graphene outer-shells served as hosts for anchoring metal atoms. By simply modulating the reduction temperature (see the preparation details in the “Methods” section) of Cu species deposited on graphitic carbon shells, we could change the dispersion state of Cu to prepare two different types of catalysts: atomically dispersed Cu catalyst (denoted as Cu_1_/ND@G) and Cu-cluster catalyst (denoted as Cu_n_/ND@G). Importantly, both of them have identical Cu loading amount (0.25 wt%). From X-ray diffraction (XRD) profiles (see Supplementary Fig. 5), no diffraction associated with bulk Cu was detected on both catalysts, demonstrating that the Cu species were highly dispersed over the substrate surface. Further structural analysis of the catalysts (see Supplementary Table 1) revealed that no obvious differences in chemical structure were found between Cu_1_/ND@G and Cu_n_/ND@G except for the dispersion of Cu species. The aberration-corrected high-angle annular dark-field scanning transmission electron microscopic (HAADF-STEM) images showed that the Cu_1_/ND@G catalyst was consisted of isolated bright spots, indicating the atomically dispersed Cu species on ND@G (Fig. 1c, d). In contrast, for Cu_n_/ND@G, the Cu species was dominated by Cu clusters, together with a small amount of atomically dispersed Cu (see Fig. 1e, f). In good agreement with the results of TEM, Cu dispersion state observed by N_2_O titration (99.8% for Cu_1_/ND@G and 85.2% for Cu_n_/ND@G, see Supplementary Table 1) further confirmed that the two catalysts, sharing the same Cu loading, have different atomic dispersion states.

**Fig. 2:**
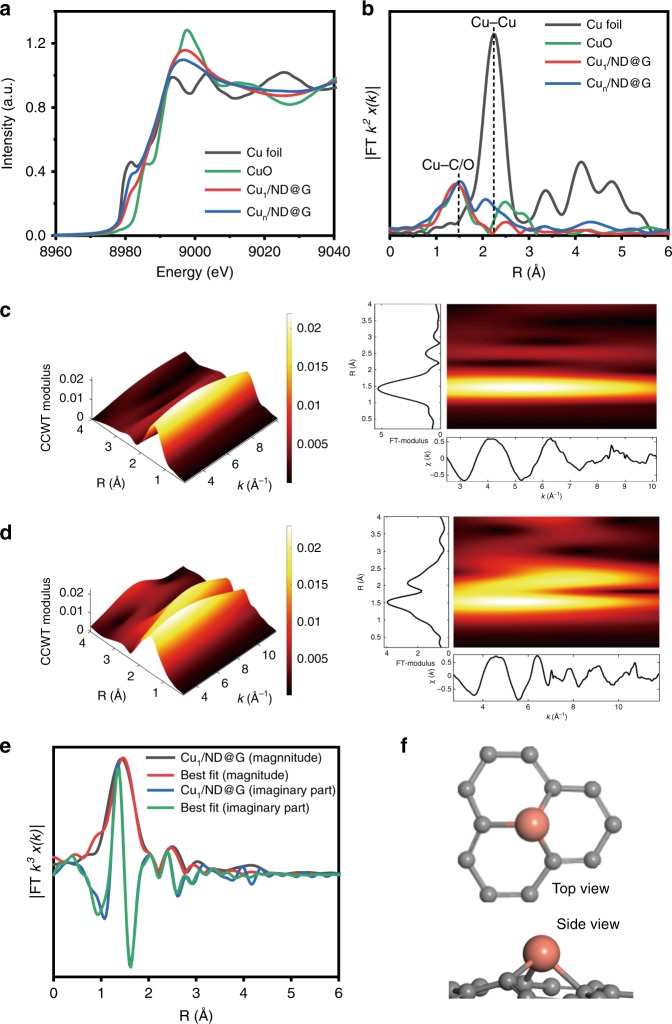
Synchrotron XAFS measurements of Cu_1_/ND@G and Cu_n_/ND@G catalysts. **a** Cu *k*-edge XANES profiles for Cu_1_/ND@G, Cu_n_/ND@G, Cu foil, and CuO. **b** Cu *k*-edge EXAFS spectra in *R* space for Cu_1_/ND@G, Cu_n_/ND@G, Cu foil, and CuO. **c** WT analysis of Cu_1_/ND@G. **d** WT analysis of Cu_n_/ND@G. **e** EXAFS fitting curve for Cu_1_/ND@G. **f** The optimized Cu-C_3_ structure; color code: Cu (orange), C (gray)

**Fig. 3:**
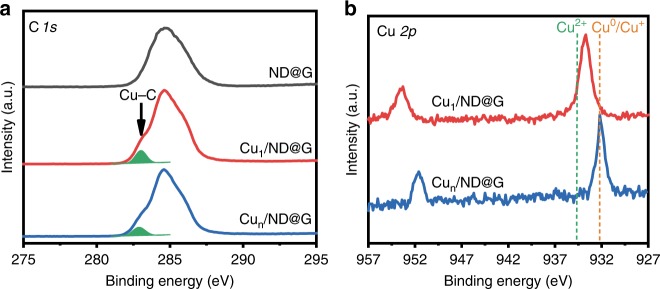
XPS measurements of ND@G support and Cu_1_/ND@G and Cu_n_/ND@G catalysts. **a** C 1*s* XPS of ND@G, Cu_1_/ND@G, and Cu_n_/ND@G. **b** Cu 2*p* XPS of Cu_1_/ND@G and Cu_n_/ND@G

The X-ray adsorption fine structure (XAFS) measurement was employed to further investigate the distinct structure of Cu species. Clearly, the near-edge feature of Cu_1_/ND@G or Cu_n_/ND@G samples was in between of those of Cu foil and CuO (Fig. 2a), indicating that the Cu species were partially positively charged (Cu^δ+^, 0 < *δ* < 2). Fourier-transformed *k*^2^-weighted extended X-ray absorption fine structure (EXAFS) in *R* space was performed to elucidate the coordination environments of Cu atoms anchored on ND@G. For Cu_1_/ND@G, the only distinct scattering was observed at 1.5 Å that corresponds to the first coordination shell of Cu-C or Cu-O. This evidences the single atom Cu on ND@G through Cu-C bonding, which is further verified by the appearance of Cu-C peak at 283 eV in C 1*s* XPS spectrum after Cu was loaded on ND@G (Fig. 3a)^32^. In contrast, for Cu_n_/ND@G, besides the scattering of Cu-C at 1.5 Å, a major peak at 2.2 Å that ascribed to Cu-Cu scattering could be observed, indicating the formation of Cu clusters. A wavelet transformation (WT) of Cu *k*-edge EXAFS oscillations also displayed the dispersion of Cu in both samples visually in both *k* and *R* spaces. Figure 2c, d are the WT contour plots of Cu_1_/ND@G and Cu_n_/ND@G that showed a Cu-C back-scattering contribution near 1.5 Å, indicating that both Cu_1_ and Cu_n_ were anchored on ND@G through the Cu-C bonding. However, as shown in Fig. 2d, another peak at 2.2 Å in Cu_n_/ND@G, which is associated with the Cu-Cu scattering, further verified the dispersion state of Cu clusters.

XPS was used to study the valence states of Cu in two catalysts (Fig. 3b). For Cu_1_/ND@G, the Cu 2*p*_3/2_ peak appeared at 933.7 eV, situated between Cu^0^/Cu^+^ (932.4 eV) and Cu^2+^ (934.6 eV)^33,34^, which is consistent with the XANES results (Fig. 2a and Supplementary Fig. 6). The results imply that the Cu species in Cu_1_/ND@G interact strongly with the substrate. Through Cu-C bonds, an elevated chemical valence of single atom Cu species due to charge transferred from Cu atoms to substrate could be observed, which was absent on Cu_n_/ND@G due to the similar properties between Cu clusters and bulk Cu.

Quantitative chemical configuration analysis of Cu_1_/ND@G and Cu_n_/ND@G were carried out through the least-squared EXAFS fitting. The *R*-space fitting results are shown in Fig. 2e and Supplementary Fig. 9, and the corresponding structure parameters are listed in Supplementary Table 2. The coordination number of the center Cu atom with surrounding C atoms on Cu_1_/ND@G was 3.1, and the mean bond length of Cu-C was 1.94 Å. Based on these results, the proposed local atomic structure of Cu was constructed as that in Fig. 2f. The isolated Cu atom was anchored over the defective sites of graphene through bonding with three C atoms.

### Acetylene hydrogenation performance over Cu_1_/ND@G and Cu_n_/ND@G

**Fig. 4:**
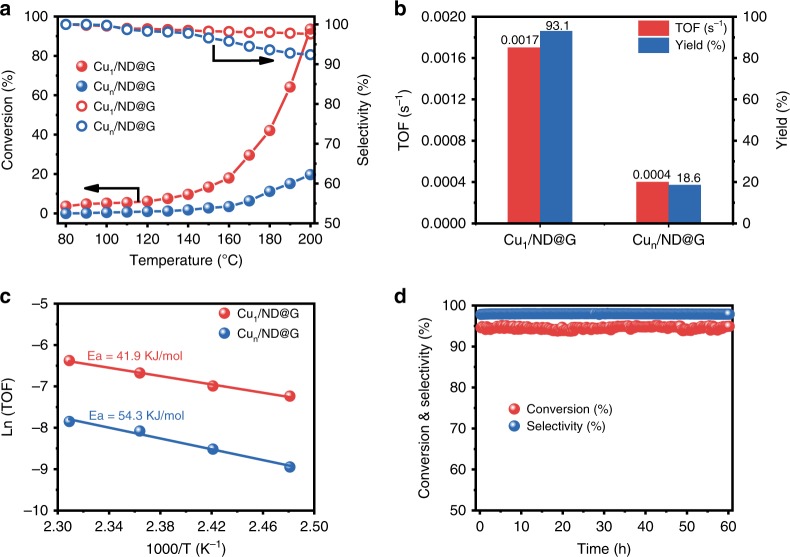
Catalytic performance of Cu_1_/ND@G and Cu_n_/ND@G. **a** Conversion and selectivity as a function of temperature for the selective hydrogenation of acetylene over the Cu_1_/ND@G and Cu_n_/ND@G catalysts. **b** TOF values (in the kinetic region) and ethylene yields (*T* = 200 °C) of the catalysts. **c** Arrhenius plots of the catalysts. **d** Durability test on Cu_1_/ND@G at 200 °C for 60 h. (reaction condition: 1% C_2_H_2_, 10% H_2_, 20% C_2_H_4_ gas mix balanced with He; GHSV = 3000 h^−1^)

Selective hydrogenation of acetylene was carried out using Cu_1_/ND@G and Cu_n_/ND@G, respectively, to gain insight into the impact of the atomic structure and spatial arrangement of Cu over the catalytic performance. The conversion and selectivity as a function of temperature over these two catalysts are shown in Fig. 4a. For aggregated Cu species in Cu_n_/ND@G, the conversion was still <20% even at 200 °C. Significantly, Cu_1_/ND@G manifested robust catalytic activity and remarkably high selectivity toward ethylene (see Fig. 4a). The conversion of acetylene reached 95% at 200 °C, with ethylene selectivity of 98%. We further compared the intrinsic activity of two catalysts, as shown in Fig. 4b. Cu_1/_ND@G showed a high TOF of 0.0017 s^−1^ (4.25 times higher than that of Cu_n_/ND@G) and a high ethylene yield of 93.1%, showing competitive advantages over former results [<90%] (see Supplementary Table 3 and Supplementary Fig. 10). Apparent activation energies (*E*_a_) of the Cu_1_/ND@G and Cu_n_/ND@G catalysts were 41.9 and 54.3 kJ/mol, respectively (see Fig. 4c), suggesting the superiority of atomically dispersed Cu catalysts. The stability of Cu_1_/ND@G catalyst was found to be excellent. As shown in Fig. 4d, the conversion and selectivity at 200 °C over Cu_1_/ND@G remained steady at 95% and 98%, respectively, for at least 60 h under reaction conditions. The atomic structure of the Cu_1_/ND@G catalyst was well maintained (see Supplementary Figs. 7–9 and Supplementary Tables 1 and 2) during the stability test. Meanwhile, under the reaction conditions where the conversion is high enough to meet the industrialization requirement (see Supplementary Fig. 11), Cu_1_/ND@G remained stable for at least 30 h.

### DFT calculations

**Fig. 5:**
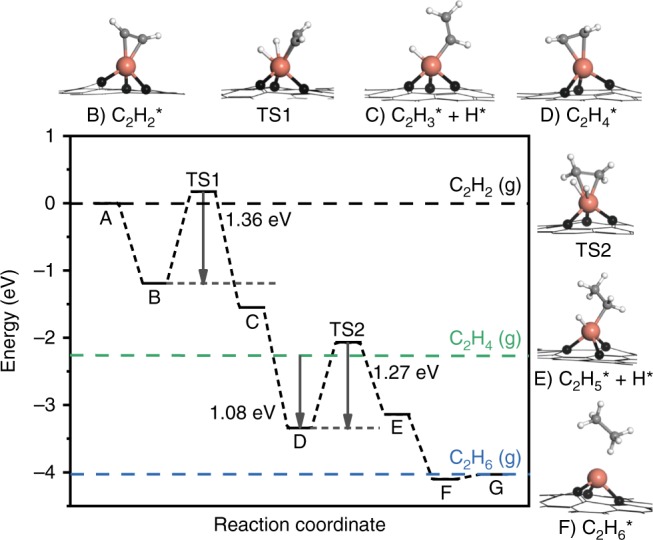
Energy profile of acetylene hydrogenation on the Cu_1_/ND@G catalyst and the structures of intermediates and transition states. Color code: Cu (orange), C in graphene (black), C in reactant/intermediates/product (gray), and H (white)

To better understand the nature of the superior acetylene hydrogenation activity of Cu_1_/ND@G, the reaction process was studied by DFT. The details of the computational simulation methods can be found in the “Methods” section. Cu_1_ supported over graphene layer (Cu_1_@Gr) was used to model the Cu_1_/ND@G catalyst, while a Cu_13_ cluster on ND@G to model the Cu_n_/ND@G catalyst. The computational details are summarized in Supplementary Information, including all of the possible binding modes of different adsorbates on the catalytic surfaces. The energy profiles (including the entropy contribution) for the catalysis of Cu_1_/ND@G are shown in Fig. 5. On Cu_1_/ND@G, the adsorption energy of acetylene on Cu atoms is −1.19 eV (see Supplementary Table 4). Then the molecular hydrogen undergoes dissociative adsorption. This step is exothermic by 0.36 eV with an energy barrier of 1.36 eV (from B to C), which is the rate determining step (RDS) for acetylene hydrogenation. On the Cu_13_ cluster catalyst, the barrier of RDS is 1.50 eV (see Supplementary Fig. 14), implying that the cluster catalyst is less active than the Cu_1_ catalyst (see Supplementary Fig. 16). More importantly, the transition-state energy of ethylene hydrogenation on Cu_1_/ND@G (TS2, 1.27 eV) is above the energy of gas-phase ethylene (1.08 eV), suggesting that ethylene favors desorption over further hydrogenation in the following step. In another word, the high selectivity of acetylene hydrogenation here is due to the priority of ethylene desorption at the atomically dispersed Cu sites of Cu_1_/ND@G. This calculated result is consistent with the observed difference in catalytic performance between Cu_1_/ND@G and Cu_n_/ND@G.

## Discussion

In summary, we synthesized an atomically dispersed Cu_1_/ND@G catalyst for acetylene semihydrogenation reaction. It exhibited remarkably outstanding acetylene conversion (~95%), ethylene selectivity (~98%), and stability (>60 h), exceeding the Cu-cluster catalyst with the same Cu loading. The unique bonding structure and electronic property of Cu atoms on Cu_1_/ND@G facilitate the acetylene activation and ethylene desorption, which clearly elucidates the importance of isolated Cu atoms in catalysts for high-performance acetylene semihydrogenation. Our results and conclusions pave the way for rational design of promising non-noble catalysts for hydrogenation processes.

## Methods

### Materials

ND powders were purchased from Beijing Grish Hitech Co., Ltd, China. Copper (II) nitrate trihydrate (Cu(NO_3_)_2_·3H_2_O) was the analytical reagent and purchased from Sinopharm Chemical Reagent Co., Ltd, China. Deionized (DI) water with the specific resistance of 18.25 MΩ cm was used in all our experiments.

### Preparation of ND@G

ND@G was prepared by annealing ND powders at 1100 °C (heating rate 5 °C min^−1^) for 4 h under flowing Ar gas (100 mL min^−1^) and then naturally cooled to room temperature. The as-prepared products were further purified by hydrochloric acid for 24 h and then washed with DI water. Finally, the ND@G nanocarbon support was obtained after drying in vacuum at 60 °C for 24 h.

### Preparation of Cu_1_/ND@G and Cu_n_/ND@G

Typically, 200 mg ND@G was dispersed into 30 mL DI water in a 100-mL round-bottom flask, and the mixture was ultrasonically treated for 30 min to obtain a homogeneous suspension. Then the pH value of ND@G support suspension was adjusted to about 11 by dropping 0.25 M Na_2_CO_3_ solution. Afterwards, 4 mL Cu(NO_3_)_2_·3H_2_O solution (containing 0.125 mg mL^−1^ Cu) was introduced into ND@G support suspension dropwise under magnetic stirring at 100 °C in oil bath and then kept stirring for 1 h. At the end, the mixture was naturally cooled to room temperature, collected by filter, washed several times with DI water, and dried in vacuum at 60 °C for 12 h.

The catalysts were reduced in H_2_ (10 vol% in He, flow rate = 50 mL min^−1^) at 200 °C for 1 h to yield Cu_1_/ND@G and at 600 °C for 1 h to obtain Cu_n_/ND@G. The catalysts after the 60-h reaction were denoted as Cu_1_/ND@G-60h and Cu_n_/ND@G-60h, respectively.

### Catalyst characterization methods

HRTEM images were taken by a FEI Tecnai G2 F20 working at 200 kV. Atomic resolution STEM images were recorded by a JEOL JEM ARM 200CF aberration-corrected cold field-emission scanning transmission electron microscope at 200 kV. XPS were carried out on ESCALAB 250 instrument with Al Kα X-rays (1489.6 eV, 150 W, 50.0 eV pass energy) and the C 1*s* peak at 284.6 eV as internal standard. XRD patterns were collected by using an X-ray diffractometer (Bruker Smart APEX II) using a Cu Kα source at a scan rate of 2° min^−1^. N_2_ physisorption were measured at −196 °C using a Micrometrics ASAP-2020 instrument. The porosity of samples was obtained through Brunauaer–Emmetr–Teller analysis with the pore volume measured at *p*/*p*_0_ = 0.99, and the pore size distribution was analyzed by BJH method from desorption branch. The dispersion of Cu species on catalysts was measured by a surface oxidation–reduction method on a AutoChem II 2920 apparatus. Typically, 200 mg sample was loaded in a quartz U-tube. After pretreatment with He at 100 °C for 30 min, the sample was reduced with 10 vol% H_2_ in Ar at 200 °C for 1 h (flow rate = 30 mL min^−1^) and cooled to 90 °C in He flow. Then 10 vol% N_2_O in He was introduced into the tube and kept for 3 h at 90 °C (flow rate = 30 mL min^−1^). The sample was purged with He again and cooled to 50 °C, and then the sample was reduced with 10 vol% H_2_ in Ar (flow rate = 30 mL min^−1^) from 50 °C to 450 °C with a heating rate of 10 °C min^−1^. Ultraviolet-Raman spectroscopy was performed on powder samples by using HORIBA LabRam HR Raman spectrometer, and the excitation wavelength was 325 nm with a power of 0.2 mW (exposure 90 s, accumulate 3 times). XAFS measurements were carried out at Shanghai Synchrotron Radiation Facility. Elemental analysis of copper in the solid catalysts was detected by inductively coupled plasma–atomic emission spectrometry (Optima 8300 DV).

### Catalytic performance tests

The selective hydrogenation activity of the catalysts was conducted in a quartz-bed flow reactor for acetylene hydrogenation with 200 mg catalysts. A gas mixture of 1 vol% C_2_H_2_, 10 vol% H_2_, and 20 vol% C_2_H_4_ with He balance (flow rate = 10 mL min^−1^, GHSV = 3000 mL g^−1^ h^−1^) was introduced, followed by ascending temperature testing. Gas chromatograph (GC) injections were done at each temperature after stabilization for 30 min. The reactants and products were analyzed by GC (Agilent 7890 A) equipped with a flame ionization detector and a HP-PLOT AL/S (HP-plot 19091 P-S15, Agilent, 50 m × 0.32 mm × 8 μm) capillary column with He as the carrier gas.

Acetylene conversion and selectivity to ethylene were calculated as the following:

1 $${\mathrm{Conversion}} = \frac{{{\mathrm{C}}_{\mathrm{2}}{\mathrm{H}}_{\mathrm{2}}\ \left({{\mathrm{feed}}} \right) - {\mathrm{C}}_{\mathrm{2}}{\mathrm{H}}_{\mathrm{2}}}}{{{\mathrm{C}}_{\mathrm{2}}{\mathrm{H}}_{\mathrm{2}}}} \times 100\%$$

2 $${\mathrm{Selectivity}} = \left(1 \, - \, \frac{{{\mathrm{C}}_{\mathrm{2}}{\mathrm{H}}_{\mathrm{6}} + {\mathrm{2C}}_4\ {\mathrm{olefin}}}}{{{\mathrm{C}}_{\mathrm{2}}{\mathrm{H}}_{\mathrm{2}}\ \left({{\mathrm{feed}}} \right) - {\mathrm{C}}_{\mathrm{2}}{\mathrm{H}}_{\mathrm{2}}}}\right) \times 100\%$$

### Computational simulations of the catalytic mechanisms by Cu_1_/ND@G and Cu_n_/ND@G

All of the catalytic structures were obtained by the geometry optimizations using the plane-wave-based DFT method implemented in the Vienna Ab Initio Simulation Package^35,36^. We describe the electron–ion interaction using the projector augmented wave method^37,38^. The generalized gradient approximation and the Perdew–Burke–Emzerhof functional^39,40^ describes the exchange and correlation energies for all systems. All the calculations take spin polarized into consideration. The plane-wave expansion of the wave functions adopted an energy cutoff of 400 eV. The Monkhorst–Pack *k*-point was set to 3 × 3 × 1 in the reciprocal lattice. The convergence criteria for electronic self-consistent interactions is 10^−5^. The geometries of bulk and surface were optimized by the conjugate gradient algorithm until the maximum force on any ion was <0.03 eV Å^−1^, where all the atoms in the catalyst and adsorbate were fully relaxed. The most stable configurations of the reactant and intermediates on Cu_1_@Gr were determined by using the climbing image nudged elastic band method^41^, and vibrational frequencies were analyzed to ensure the transition state with only one imaginary frequency.

## Supplementary information

supplementary information

Peer Review

Source Data

## Data Availability

The data supporting this article and other findings are available from the corresponding authors upon request. The source data underlying Figs. 2a, b, e, 3a, b, 4a–d, and 5 and Supplementary Figs. 2a, b, 3a–c, 4a–f, 5, 6, 7a, b, 8a, b, 10, 11, 14, 15, and 16 are provided as a Source Data file.
