# Supplementary material for: Anchoring Cu1 species over nanodiamond-graphene for semi-hydrogenation of acetylene
Source: Nat Commun. 2019 Sep 30;10:4431. doi: 10.1038/s41467-019-12460-7 (PMC6768864; doi:10.1038/s41467-019-12460-7)
Supplement: Supplementary file 1 — supplementary information [file 41467_2019_12460_MOESM1_ESM.pdf]

Supplementary Information

Anchoring Cu_1 species over nanodiamond-graphene for semi-hydrogenation of acetylene

Huang et al.

Supplementary Figures and Tables

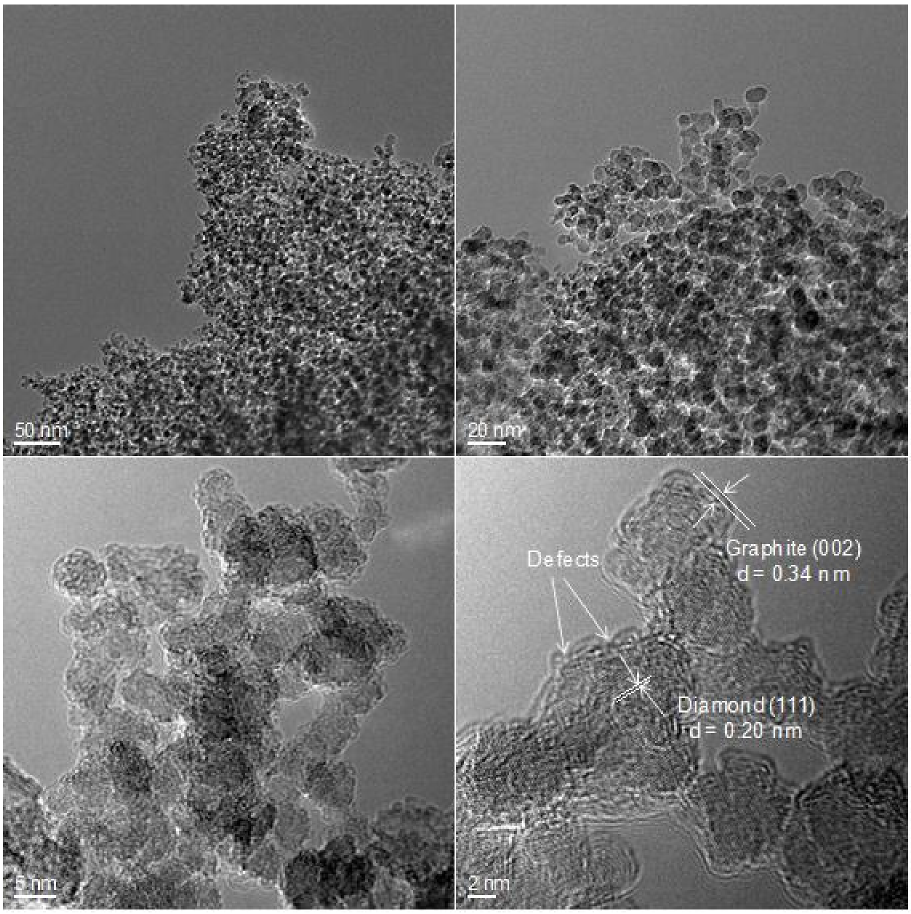

Supplementary Figure 1. HRTEM images of ND@G .

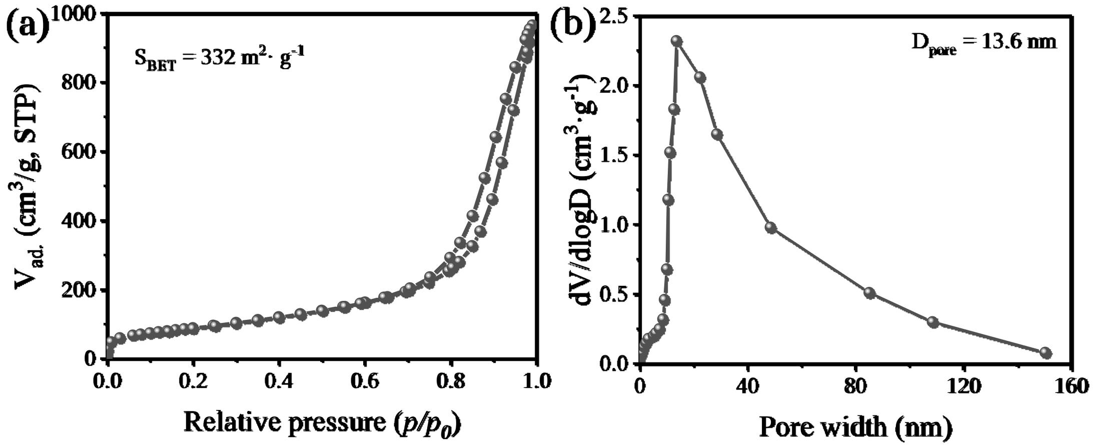

Supplementary Figure 2. N_2 adsorption-desorption isotherm (a) and BJH pore size distribution (b) of ND@G.

Supplementary Table 1. Physical structure properties of the catalysts

Samples	Cu loading (wt %)		Cu dispersion[b]	S_{BET}[c]	V_{pore}[d]	D_{pore}[e]
	Theoretical	Measured[a]	(%)	($m^2 \cdot g^{-1}$)	($cm^{-3} \cdot g^{-1}$)	(nm)
Cu_1/ND@G	0.25	0.23	99.8	335	1.50	13.6
Cu_n/ND@G	0.25	0.23	85.2	352	1.52	12.8
Cu_1/ND@G-used	0.25	0.22	99.8	332	1.50	13.2

[a]determined by ICP-AES; [b]determined by N_2O titration; [c]BET method; [d]Volume of N_2 at p/p_0 = 0.98; [e]N_2 desorption.

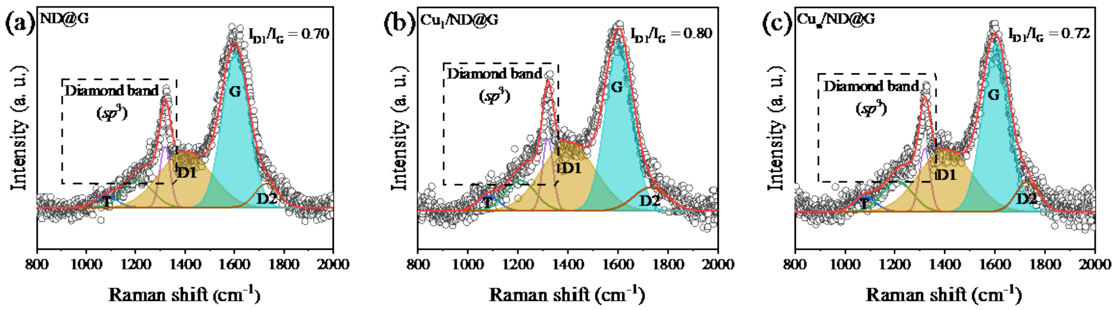

Supplementary Figure 3. Raman spectra of (a) ND@G, (b) Cu_1/ND@G, (c) Cu_n/ND@G. (The weak peak at 1080 cm^{-1} was assigned to C-C sp^3 vibration (T); The broad peak at 1210 cm^{-1} and the sharp peak at 1318.7 cm^{-1} together belongs to diamond band; The broadest peak at approximately 1400 cm^{-1} assigned to the disorder-induced D band (D1); The strongest peak at 1603 cm^{-1} could be assigned to the well-ordered nanocrystalline graphite (G) and a shoulder peak to this peak at 1730 cm^{-1} assigned to C=O stretching vibration (D2); I_{D1}/I_G (where I_{D1} and I_G are the integral areas values of D1-band and G-band) are used to qualitatively evaluate the defect degree of the samples)[1].

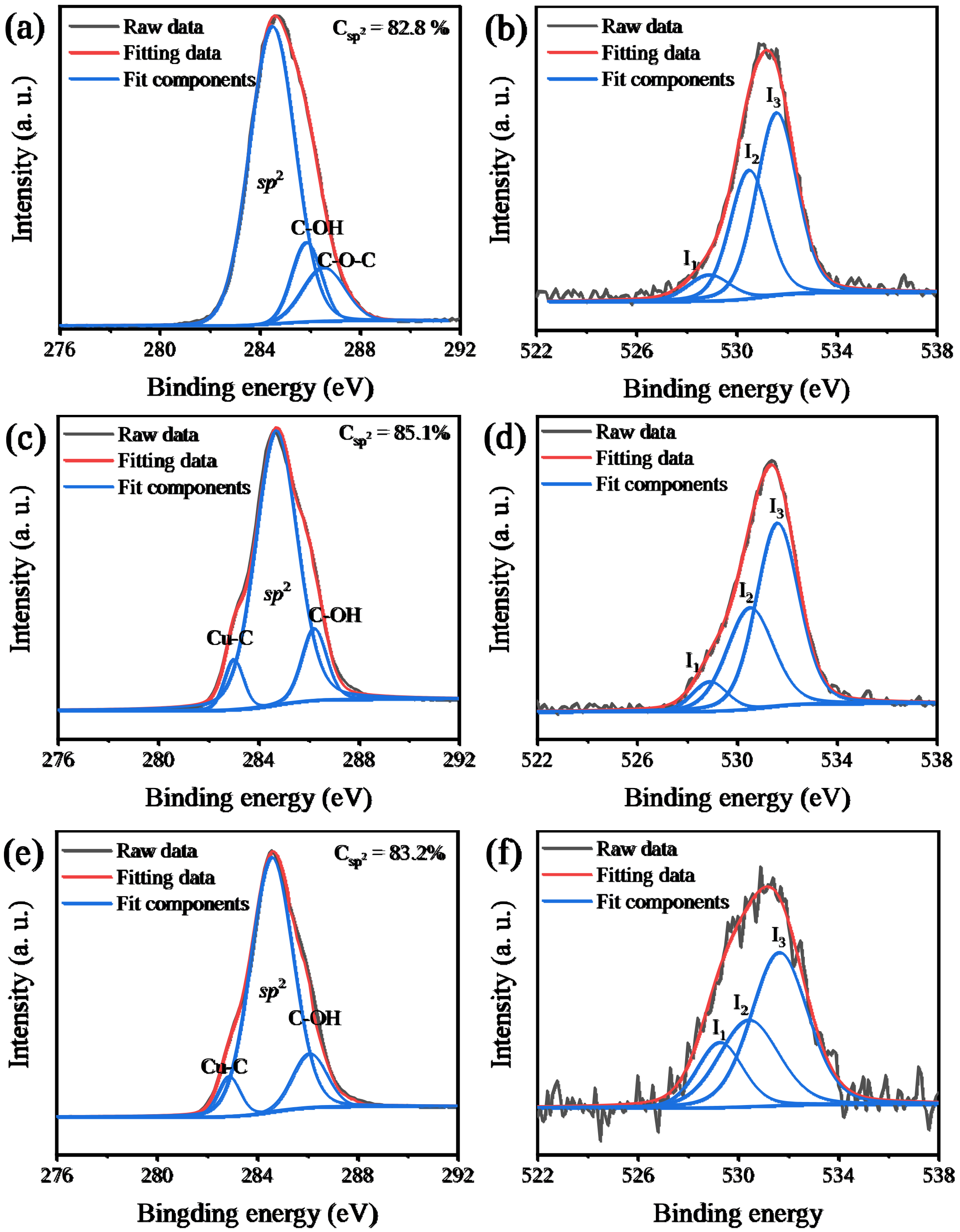

Supplementary Figure 4. XPS spectra for the (a) C 1*s*, and (b) O 1s regions of ND@G; XPS spectra for the (c) C 1*s*, and (d) O 1*s* regions of Cu_1/ND@G; XPS spectra for the (e) C 1*s*, and (f) O 1*s* regions of Cu_n/ND@G. (The XPS peaks were fitted to 20% Lorentzian-Gaussian character, after performing a Shirley background subtraction. The sp^2 peak of the C 1s envelope centered at 284.5 eV had a fwhm of 1.8 eV. I_1 denotes C=O (oxygen doubly bonded to aromatic carbon); I_2 denotes C-O (oxygen singly bonded to aliphatic carbon); I_3 denotes phenolic (oxygen singly bonded to aromatic carbon).)[2].

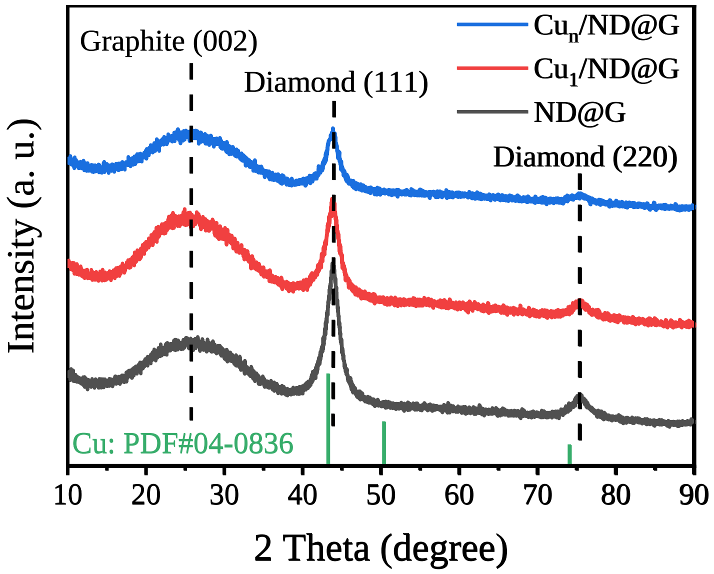

Supplementary Figure 5. XRD patterns of ND@G, Cu_1/ND@G and Cu_n/ND@G.

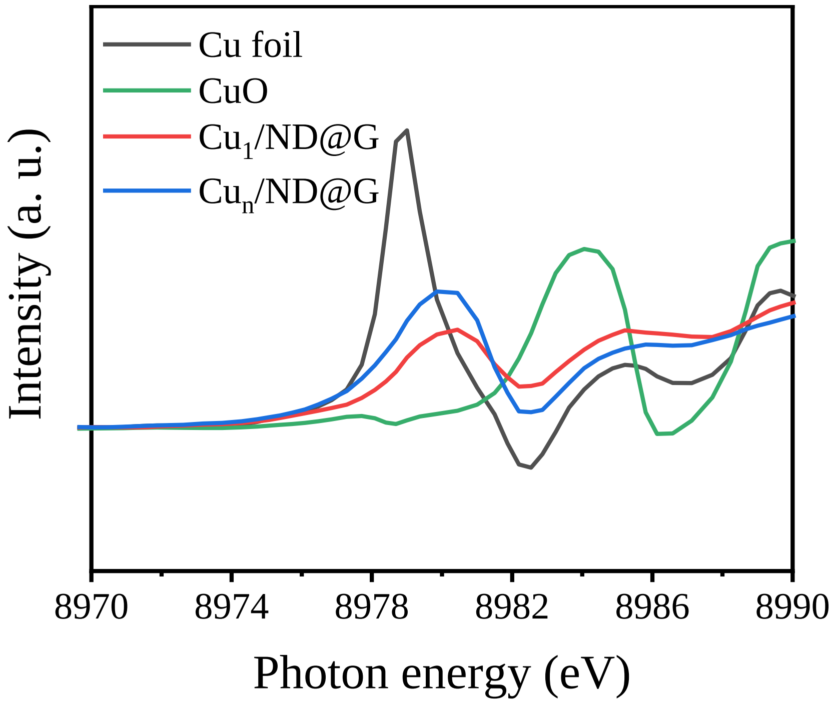

Supplementary Figure 6. First derivative of normalized intensity of Cu K-edge XANES profiles.

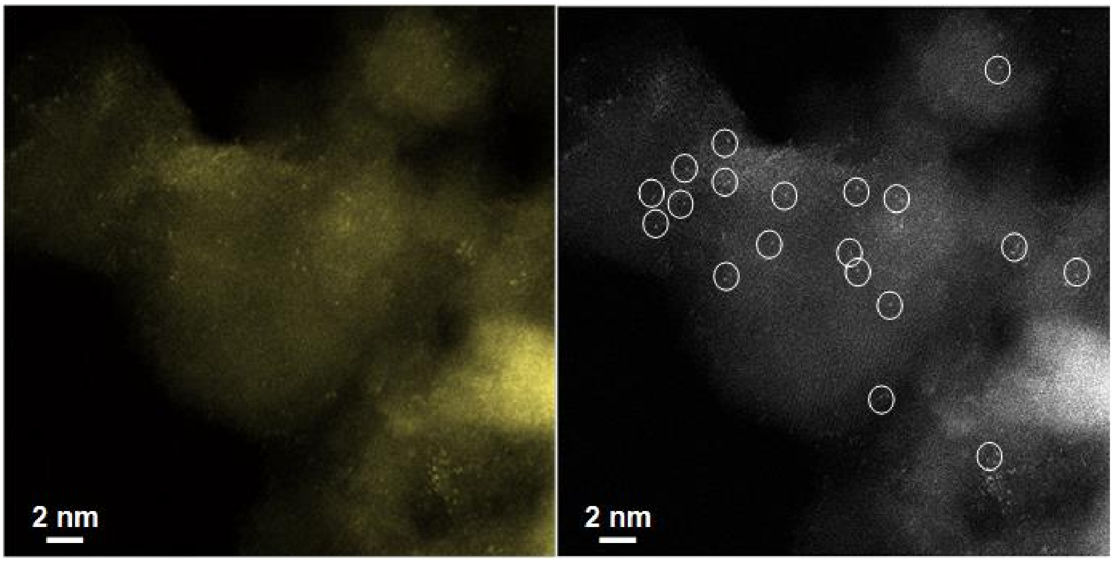

Supplementary Figure 7. HADDF-STEM images of Cu_1/ND@G-60h.

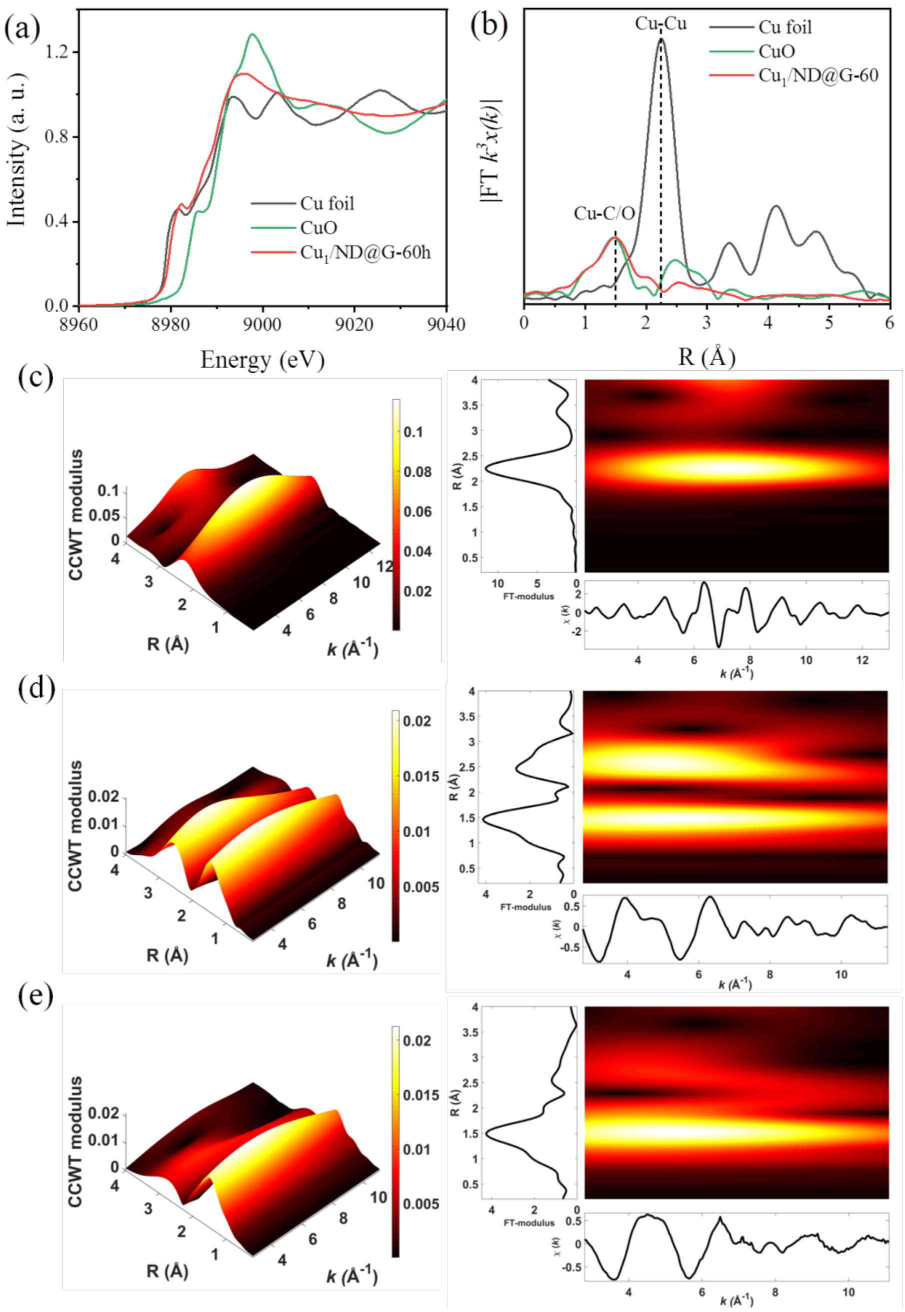

Supplementary Figure 8. Cu K-edge XANES profiles (a) for Cu_1/ND@G-60h, Cu foil and CuO; Cu K-edge EXAFS spectra in R space (b) for Cu_1/ND@G-60h, Cu foil and CuO; WT analysis of (c) Cu foil, (d) CuO, and (e) Cu_1/ND@G-60h.

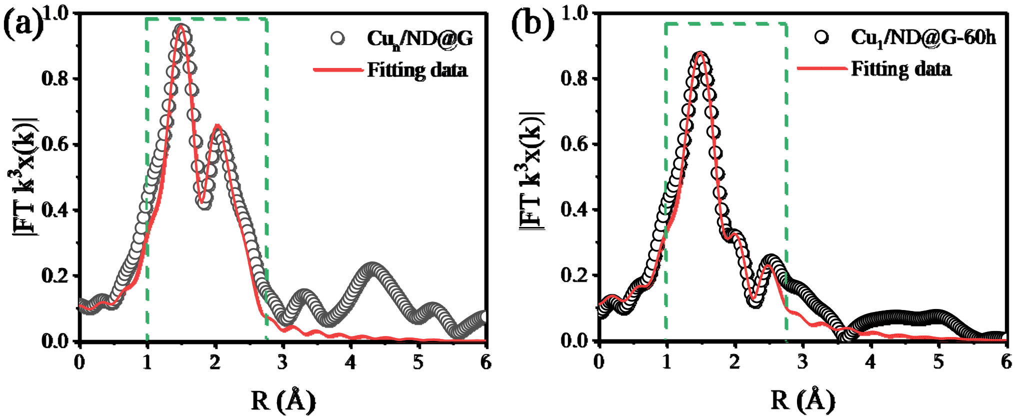

Supplementary Figure 9. EXAFS fitting curve for (a) Cu_n/ND@G and (b) Cu_1/ND@G-60h.

Supplementary Table 2. Structural parameters extracted from quantitative EXAFS curve-fitting.

Samples	Path	C.N.[a]	R (Å)[b]	σ^2 (10^{-3}Å^2)[c]	E_0 (eV)[d]	R factor (%)
Cu_1/ND@G	Cu-C/O	3.1	1.94 ± 0.02	3.8	1.1	0.4
	Cu-Cu	N.A.	-	-	-	
Cu_n/ND@G	Cu-C/O	2.4	1.89 ± 0.02	2.9	7.0	2.0
	Cu-Cu	2.2	2.51 ± 0.03	9.3	-2.0	
Cu_1/ND@G-60h	Cu-C/O	2.8	1.90 ± 0.03	5.1	8.7	0.5
	Cu-Cu	N.A.	-	-	-	

[a]C.N. is the coordination number; [b]R is interatomic distance (the bond length between Cu center atoms and surrounding coordination atoms); [c]σ^2 is Debye-Waller factor (a measure of thermal and static disorder in absorber scatter distances); [d]E_0 is edge energy shift (the difference between the zero kinetic energy value of the sample and that of theoretical model).

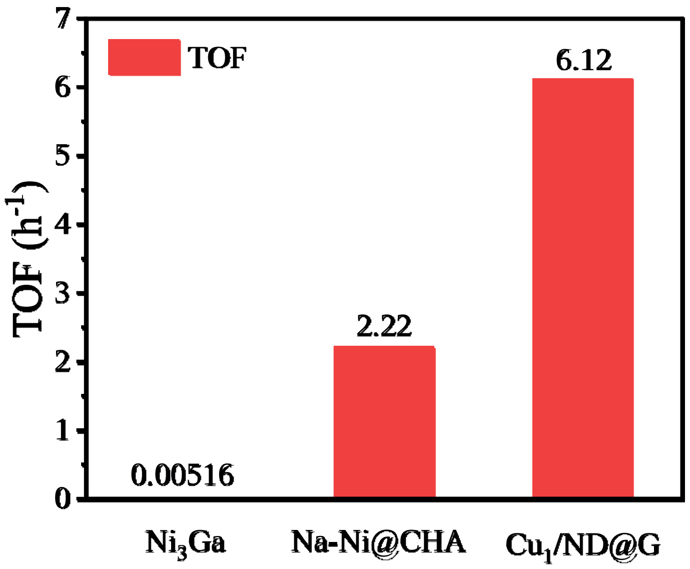

Supplementary Figure 10. TOF values of non-noble metals for acetylene for acetylene hydrogenation (T = 80 ^{o}C; at below 20% acetylene conversion, in the kinetic region; The figure was created by us based on the cited data. The data of Ni_3Ga and Na-Ni@CHA in x-axis are from supplementary reference 3 and 4, respectively. The data of Cu_1/ND@G in x-axis is from our work.).

Supplementary Table 3. Catalytic performance of different non-noble metals based acetylene hydrogenation.

catalyst	Metal loading (%)	T (^oC)	Feed (C_2H_2:H_2:C_2H_4)	Conversion (%)	Selectivity (%)	Ref.
$[Fe^{III}-(H_2O)_6][Fe_2^{III}(\mu-O)_2(H_2O)_6]_{1/2}\{Ni^{II}_4[Cu^{II}_2(Me_3mpba)_2]_3\}\cdot 72H_2O$	2.8	150	1 mL/min (1.2% C_2H_2/C_2H_4); 2 mL/min H_2	100	< 90	5
Fe^{III}-nZrO_2	10	150		90	> 85	
nFe_2O_3-nTiO_2	0.5	200		100	90	
$Al_{13}Fe_4$	-	200	0.5%:5%:50%	~95	81-84	6
Cu_1/ND@G[a]	0.25	200	1%:10%:20%	>95	>98	This work
Cu_1/ND@G[b]	0.25	180	1%:10%:20%	>99.95	>96	
Na-Ni@CHA	3.5	180	0.5%:8%:50%	100	90	4
Ni SAc/N-C	-	200	0.5%:10%:50%	90	90	7
Ni_3Ga	-	200	0.5%:10%:50%	90	77	3

a. GHSV = 3000 h^{-1}; b. GHSV = 2400 h^{-1}. The data of row 1, 2 and 3 are obtained from supplementary reference 5; The data of raw 4 is from supplementary reference 6; The data of row 5 and 6 are from our work; The data of raw 7, 8 and 9 are obtained from supplementary reference 4, 7 and 3, respectively.

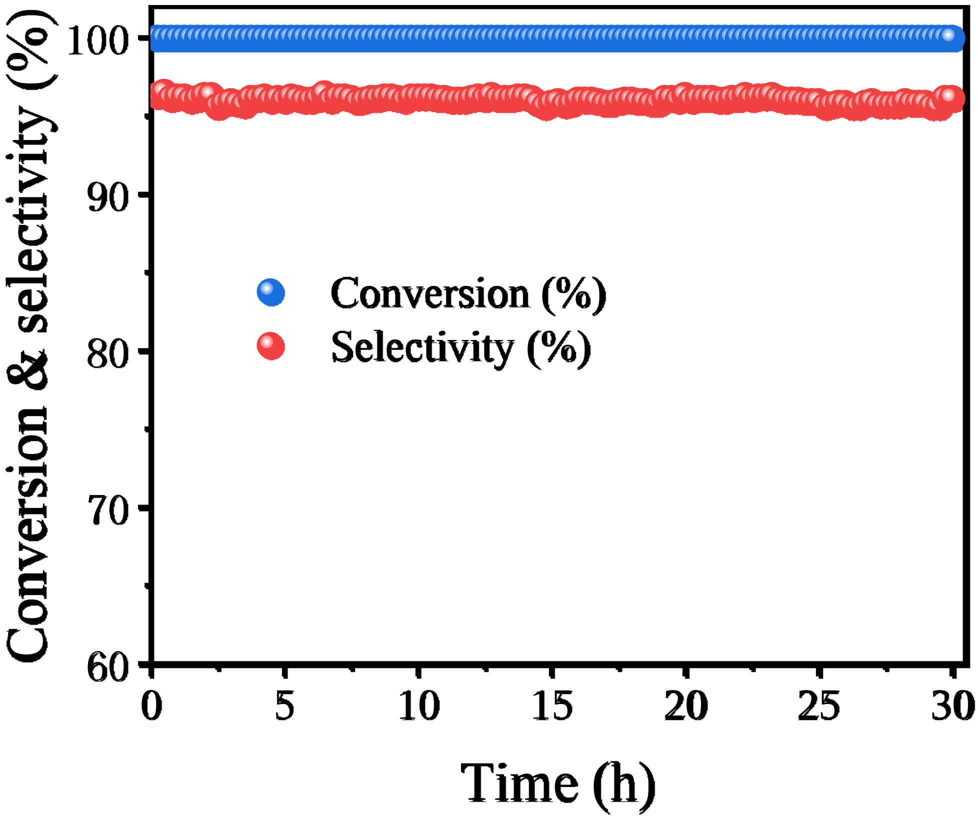

Supplementary Figure 11. Durability test on Cu_1/ND@G at 180 °C with 100% acetylene conversion and 95% ethylene selectivity for 30 h. (Reaction condition: 1% C_2H_2, 10% H_2, 20% C_2H_4 gas mix balanced with He; GHSV = 2400 h^{-1}.)

Supplementary Table 4. Reaction energies (E_r, eV) and barriers (E_a, eV) for acetylene hydrogenation on Cu_1@Gr.

Number	Reaction	E_r (eV)	E_a (eV)
1	C_2H_2(g) = C_2H_2*	-1.19	-
2	H_2(g) = H_2*	-0.29	-
3	C_2H_2* + H_2(g) = C_2H_3* + H*	-0.36	1.36
4	C_2H_3* +H* = C_2H_4*	-1.79	0.10
5	C_2H_4*= C_2H_4(g)	1.08	-
6	C_2H_4* + H_2(g) = C_2H_5* + H*	0.20	1.27
7	C_2H_5* + H* = C_2H_6*	-0.96	0.13
8	C_2H_6* = C_2H_6(g)	0.07	-

Supplementary Table 5. The bond length (Å) of key intermediates during acetylene hydrogenation on Cu_1@Gr.

Intermediate	Bond length (Å)				
	C-C(C_2Hx)	C(C_2H_x)-Cu	H(H_2)-Cu	H-H(H_2)	C-H(H_2)
C_2H_2*	1.26	1.97	-	-	-
TS1	1.28	2.10	1.65	1.06	1.44
C_2H_4*	1.40	2.08	-	-	-
TS2	1.44	2.15	1.63	1.22	1.43

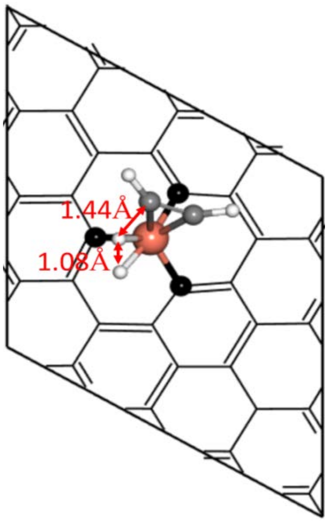

Supplementary Figure 12. The activation transition states (TS1) of the first H_2 gas molecule on Cu_1@Gr. Color code: Cu (orange), C in graphene (black), C in reactant/intermediates/product (gray) and H (white).

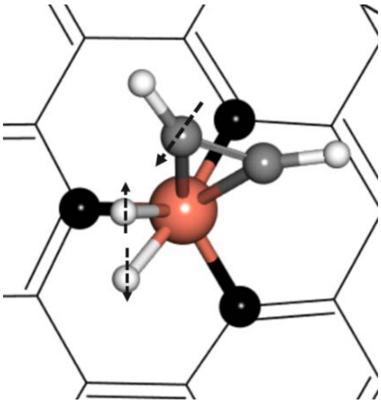

Supplementary Figure 13. The vibration direction of the only imaging frequency of TS1 on Cu_1@Gr. (Energy of the only imaging frequency is 160 meV).

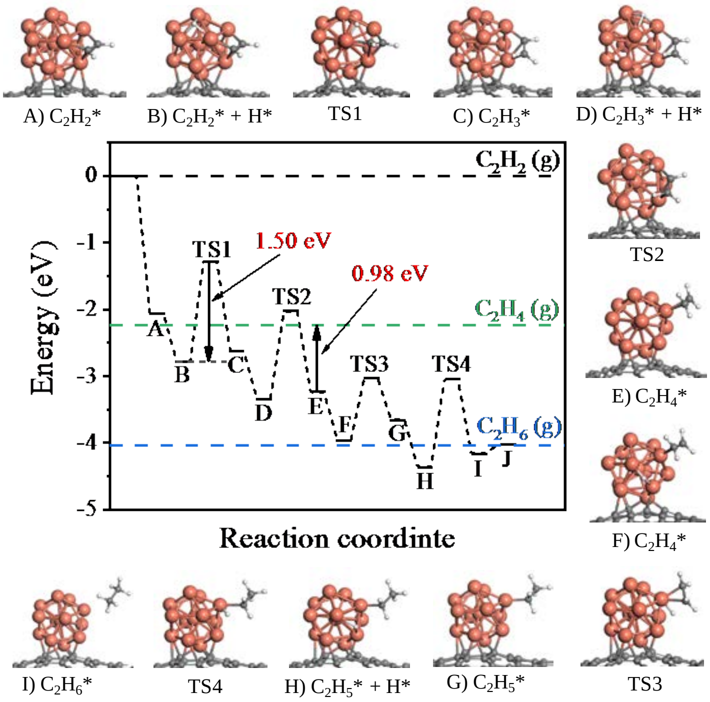

Supplementary Figure 14. Reaction profile for acetylene hydrogenation on Cu_{13}@Gr.

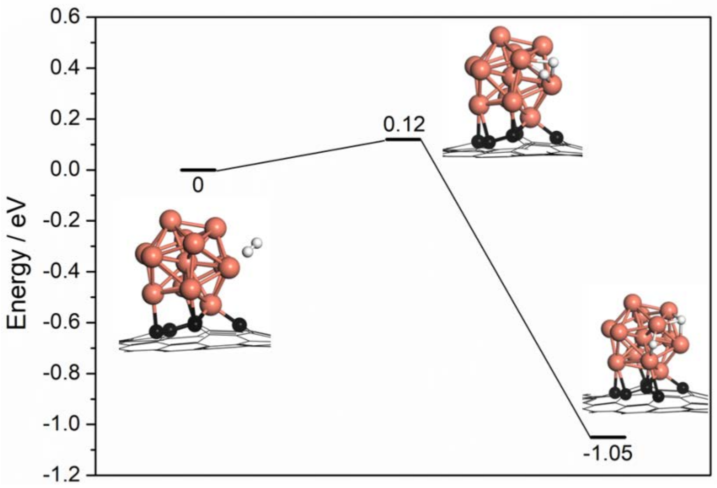

Supplementary Figure 15. The energy barrier of H_2 activation on Cu_{13}@Gr.

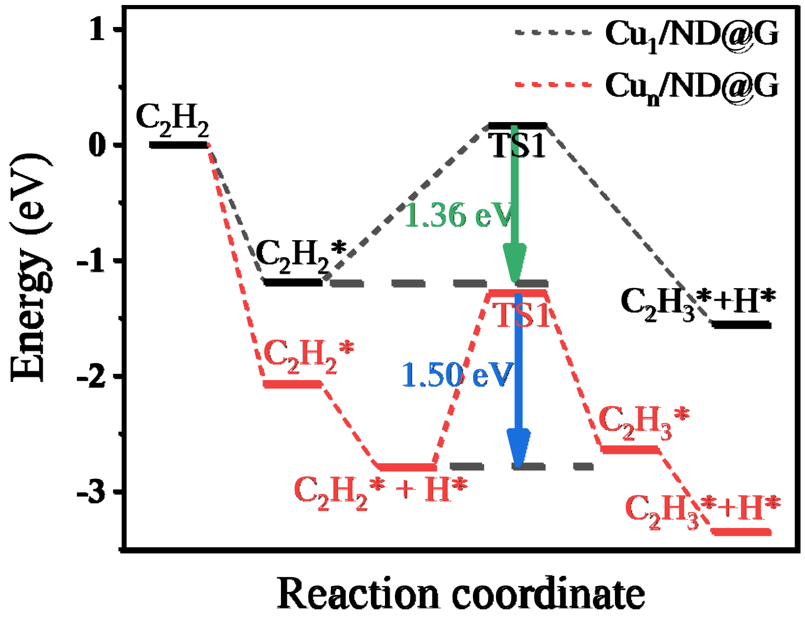

Supplementary Figure 16. The comparison of energy barrier for acetylene hydrogenation over Cu_1@Gr and Cu_{13}@Gr.

Supplementary Table 6. Reaction energies (E_r, eV) and barriers (E_a, eV) for acetylene hydrogenation on Cu_{13}@Gr.

Number	Reaction	E_r (eV)	E_a (eV)
1	$C_2H_2(g) = C_2H_2*$	-2.06	-
2	$C_2H_2* + H* (g) = C_2H_3*$	0.15	1.50
3	$C_2H_3* +H* = C_2H_4*$	0.10	1.31
4	$C_2H_4* = C_2H_4(g)$	0.98	-
5	$C_2H_4* + H* = C_2H_5*$	0.31	0.93
6	$C_2H_5* + H* = C_2H_6*$	0.20	1.32
7	$C_2H_6* = C_2H_6(g)$	0.13	-

Supplementary Table 7. The adsorption energies (ΔE, eV) of possible acetylene adsorption configurations on Cu_{13}@Gr.

Configurations	Structures	ΔE, eV
1	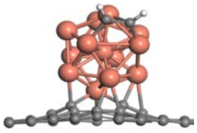	-505.69
2	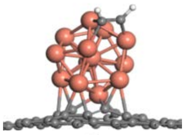	-505.61
3	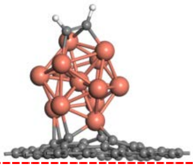	-505.43
**4**	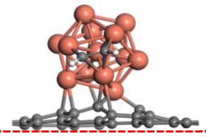	**-506.23**
5	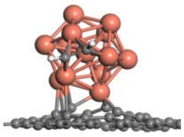	-506.01
6	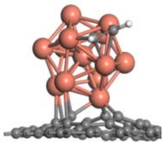	-506.08
7	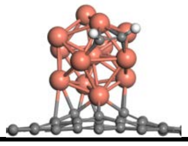	-506.05

Configuration 4 is the most stable adsorption configuration of acetylene on Cu_{13}@Gr.

Supplementary Table 8. The adsorption energies (ΔE, eV) of possible hydrogen adsorption confrigurations on Cu_1/ND@G.

Configurations	Structures		ΔE, eV
	Top view	Side view	
**H_2-Phys**	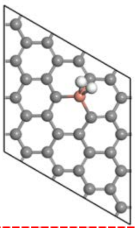	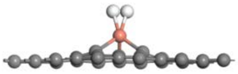	**-0.29**
H_2-Chem-a	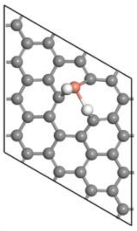	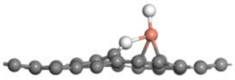	-0.28
H_2-Chem-b	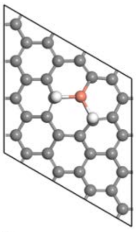	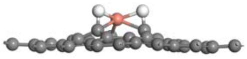	-0.28
H_a	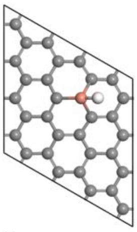	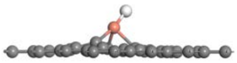	-0.02
H_b	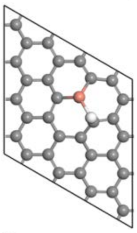	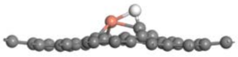	-0.27
H_c	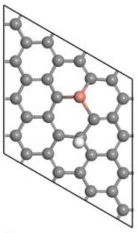	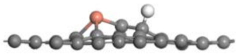	-1.01
H_d	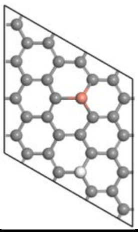	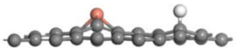	-1.33

H_2-Phys is the most stable adsorption configuration of hydrogen on Cu_1@Gr.

Supplementary reference

(1) Wang, R., Sun, X., Zhang, B., Sun, X. & Su, D. Hybrid nanocarbon as a catalyst for direct dehydrogenation of propane: formation of an active and selective core-shell sp^2/sp^3 nanocomposite structure. *Chem.-Euro. J.* **20**, 6324-6331 (2014).

(2) Ganguly, A., Sharma, S., Papakonstantinou, P. & Hamilton, J. Probing the thermal deoxygenation of graphene oxide using high-resolution in situ X-ray-based spectroscopies. *J. Phys. Chem. C* **115**, 17009-17019 (2011).

(3) Liu, Y. et al. Intermetallic Ni_xM_y (M = Ga and Sn) nanocrystals: a non-precious metal catalyst for semi-hydrogenation of alkynes. *Adv. Mater.* **28**, 4747-4754 (2016).

(4) Chai, Y. et al. Acetylene selective hydrogenation catalyzed by cationic nickel confined in zeolite. *J. Am. Chem. Soc.* **141**, 9920-9927 (2019).

(5) Tejeda-Serrano, M. et al. Isolated Fe(III)-O sites catalyze the hydrogenation of acetylene in ethylene flows under front-end industrial conditions. *J. Am. Chem. Soc.* **140**, 8827-8832 (2018).

(6) Armbrüster, M. et al. $Al_{13}Fe_4$ as a low-cost alternative for palladium in heterogeneous hydrogenation. *Nature Mater.* **11**, 690-693 (2012).

(7) Dai, X. et al. Single Ni sites distribution on N-doped carbon for selective hydrogenation of acetylene. *Chem Commun.* **53**, 11568-11571 (2017).
