# Supplementary material for: Anchoring Cu1 species over nanodiamond-graphene for semi-hydrogenation of acetylene
Source: Nat Commun. 2019 Sep 30;10:4431. doi: 10.1038/s41467-019-12460-7 (PMC6768864; doi:10.1038/s41467-019-12460-7)
Supplement: Supplementary file 2 — Peer Review [file 41467_2019_12460_MOESM2_ESM.pdf]

Reviewers' comments:

Reviewer #1 (Remarks to the Author):

In their manuscript, the authors claim an outstanding performance of a novel Cu1-based catalyst in the selective hydrogenation of alkynes. While this subject and the author's conclusions may be of broad scientific interest, it seems like large parts of the manuscript are missing.
As such, the experiments on the catalytic activity, even though included in Figure 3, are not discussed in the text. Also a section about DFT studies on Cu13-clusters seems to be missing. I can, however, only guess at the existence of the latter from the TOC-graphic and SI, since it is not mentioned anywhere in the manuscript text.

Before the missing parts of the manuscript are not included, it is not possible to assess the validity of this study. I do not recommend publication before these major gaps are covered.

If the authors consider including the above mentioned parts, there are further issues to address:
1. The performance of ceria in the alkyne hydrogenation reaction has recently been under serious debate. For a balanced account, I suggest to also refer to controversial studies. (e.g. Cao, T.; You, R.; Zhang, X.; Chen, S.; Li, D.; Zhang, Z.; Huang, W., An in situ DRIFTS mechanistic study of CeO2-catalyzed acetylene semihydrogenation reaction. PCCP 2018, 20 (14), 9659-9670, Padole, M. C.; Gangwar, B. P.; Pandey, A.; Singhal, A.; Sharma, S.; Deshpande, P. A., Adsorption of C2 gases over CeO2-based catalysts: synergism of cationic sites and anionic vacancies. PCCP 2017, 19 (21), 14148-14159).
2. Fig. 1.: Please indicate scale in images.
3. Figures S3 and S4: What is the origin of the C-O and C=O bands?
4. Figure 2 is very difficult to read. Please provide higher quality images with larger font. Consider splitting the figure in two and increasing the size.
5. line 153: "coordination number […] was 3.1 Å" This seems to be a mistake.
6. DFT section: Please describe binding modes.
7. DFT section: It is unclear where H2 dissociates. Please clarify.

Reviewer #2 (Remarks to the Author):

In this work, the authors report two new hybrid materials where atomically dispersed copper (Cu) or clusters catalysts are supported on a defective nanodiamond-graphene. Only in brief with Figure 3 report on catalytic activity of such materials, tested for the selective hydrogenation of acetylene to get ethylene.

Although the manuscript includes structural characterization and computation studies of materials, in my opinion, this work does not represent the requisite conceptual or functional leap for publication in Nature Communications. The obtained materials have not been demonstrated, neither a good reproducibility or to provide a functional benefit, and the performances, claimed but not demonstrated, for what should be the better catalyst Cu1/ND@G , is in line with a great deal of previous work on nontoxic and inexpensive metals. While the structural study is indeed interesting but not enough, the work seems more suitable for publication elsewhere after revisions. Some specific comments follow.

The structural feature are defined unique even if literature say the contrary.
The discriminant factor to prepare Cu1/ND@G versus Cun/ND@G is just temperature at which reduction via H2 is performed, 200 °C or 600 °C for 1 hour respectively. My feeling is that synthetic pathway is not well defined and/or reproducible.
To discard mixed valence states it could helpful electronic paramagnetic resonance (EPR) measurements.

Authors started saying in intro that relatively high temperature are needed in case of Ceria or Fe oxide while measuring activity at 200 °C, it is demonstrated the contrary, in fact Fe(III) does work at 150°C and with an higher GHSV.
Even if authors spent 80% of the text trying to define structures, with a very poor report on catalytic activity, no any study on structure before and after the continuous hydrogenation of acetylene in an ethylene flow at working temperature has been reported.
Most, if not all, of the figures related to TEM characterization in the manuscript seems of poor resolution. In several cases this makes deciphering the structural nuance of these materials and the key structural transition difficult. What I mean is that it could be possible a different percentage of copper clusters but it cannot be discarded in Cu1/ND@G . Also EXAFS in Fig.2b shows a shoulder on red line in the region of Cu-Cu distances which may suggest a presence of copper clusters as well.

A fastidious "superlative writing" pervades the whole paper where the authors repeatedly use the terms "excellent, unique, outstanding" and so on. Never I could read something like "To the best of our knowledge"
The authors repeatedly state that the material shows "remarkably high selectivity" or exhibits "good stability", without providing any sort of metric that makes this true (Figure 3 is not enough). One could argue the contrary as no comparisons with other inexpensive and relatively nontoxic metals such as Fe, Ti, or Zr, is discussed.

I simply do not find any aspect of the present paper really exceptional though.

Reviewer #3 (Remarks to the Author):

This manuscript reports the synthesis, characterization and catalytic performance during the semi-hydrogenation of acetylene in ethylene flows of Cu1 or Cun (n>1 and up to 20?) anchored on a defective nanodiamond/graphene material. The catalytic material is really interesting and the characterization part is impeccable, which firmly supports the proposed reaction mechanism together with DFT calculations. However, two major concerns regarding the superiority of this catalyst compared to previous ones arise:
1- Nanodiamonds are of course far of being "cheap and earth-abundant" materials, thus undermining the advantage of using Cu as a catalytic metal instead of, i.e., Pd. Notice that the support constitutes >99 wt% of the material and that the commercial catalyst is made of simple alumina. Please also notice that solid catalysts with expensive metals are generally recycled after a catalyst lifetime which minimizes losses of the expensive metal. The case here resembles to me that of "earth-abundant" metals with extremely expensive and difficult to prepare ligands in organometallic chemistry. These comments do not underestimate the academic value of the results here presented with Cu1 and the potential expansion of these results to other more available supports, however, the particularity of the nanodiamond/graphene material for a real application must be accepted.
2- The industrial semi-hydrogenation of acetylene under front-end conditions (those here simulated) operates, at least, at >99.9% conversion and >99% selectivity. In the industrial process, acetylene must be converted from 1 mol% to <10 parts-per-million, otherwise the subsequent catalyst for polyethylene manufacture is poisoned, and potential explosive acetylide species can be formed. This requisite is not arguably but a "must" for any catalyst for semi-hydrogenation of acetylene. In the other hand, the formation of ethane affects to the process "only" in economic terms, and its value can be more flexible in academic works. Thus, the 95% selectivity here reported is still acceptable, but not the 98% conversion. The reaction must be run in order to get >99.9% conversion, and then see if the Cu1 catalyst can still keep a 95% selectivity.
The manuscript is in general well-written and well-presented, however, I cannot find the paragraph commenting the catalytic results (Figure 3 and some Figures in the SI). The paragraph may have been skipped during the manuscript production and the reader can only interpret the results from the

graphics. Other minor typos: Page 7, line 111 “both catalysts”; P13, L209 “catalysts”; P14, L219 “º C”, “were measured”, L226 “cooled”, P15, L243 “calculated”.

Response to Reviewers

Reviewer #1

Comments: In their manuscript, the authors claim an outstanding performance of a novel Cu_1-based catalyst in the selective hydrogenation of alkynes. While this subject and the author's conclusion may be a broad scientific interest, it seems like large parts of the manuscript are missing.

As such, the experiments on the catalytic activity, even though included in Figure 3, are not discussed in the text. Also a section about DFT studies on Cu_{13}-clusters seems to be missing. I can, however, only guess at the existance of the latter from the TOC-graphic and SI, since it is not mentioned anywhere in the manuscript text.

Before the missing parts of the manuscript are not included, it is not possible to assess the validity of this study. I do not recommend publication before these major gaps are covered.

Response: We wholeheartedly thank the reviewers for spotting the mistakes (such content was deleted by accident) in our original submission. A similar point was raised by Reviewers 2 and 3 as well. In this revised version, we added a few paragraphs in the main text to discuss the catalytic activities (see the paragraphs in blue on Page 10, Line 15 to Page 11, Line 18).

We also added the details for the DFT simulations to the Methods section. We also expanded the discussion of the DFT simulation results in the main text (see the paragraph in blue on Page 12, Line 5 and Page 13, Line 2).

Comment 1. The performance of ceria in the alkyne hydrogenation reaction has recently been under serious debate. For a balanced account, I suggested to also refer to controversial studies. (e.g. Cao, T.; You, R.; Zhang, X.; Chen, S.; Li, D.; Zhang, Z.; Huang, W., An in situ DRIFTS mechanism study of CeO_2-catalyzed acetylene semihydrogenation reaction. PCCP 2018, 20 (14), 9659-9670, Padole, M. C.; Gangwar, B. P.; Pandey, A.; Singhal, A.; Sharma, S.; Deshapande, P. A., Adsorption of C_2 gases over CeO_2-based catalysts: sunergism of cationic sites and anionic vacancies. PCCP 2017, 19 (21), 14148-14159).

Response: Following the reviewer's suggestion, we cited these recent progress using ceria for alkyne hydrogenation [by Huang, W. (PCCP 2018, 20 (14), 9659-9670), Deshpande, P. A. (PCCP 2017, 19 (21), 14148-14159) as **Ref. 15** and **Ref. 16**.

Comment 2. Fig. 1: Please indicate scale in images.

Response: We thank the reviewer for the suggestion. However, papers published in Nature Communications are recommended to place their scale of images in figure legends. Thus, according to the format requirements of Nature Communications, we have indicated the scale of images in the legend of the Figure 1 but not marked in the images.

Comment 3. Figures S3 and S4: What is the origin of the C-O and C=O bands?

Response: Actually, they come from residual oxygen over the surface of nanodiamonds. Nanodiamond powders were synthesized by the detonation expensive method followed by acid washing for purification. After acid washing, substantial amount of oxygen-containing functional groups are produced on the surface of nanodiamonds. After annealing at 1100 ^{o}C, some relatively stable oxygens still exist on the bent graphene-like surface of ND@G. The relevant details have been discussed in our previous publications (Angew. Chem. Int. Ed. **2010**, *49*, 8640 −8644; Chem. Eur. J. **2014**, *20*, 6324 – 6331).

Comment 4. Figure 2 is very difficult to read. Please provide higher quality images with larger font. Consider splitting the figure in two and increasing the size.

Response: We thank the reviewer for pointing this out for us. we split the original Figure 2 into two parts (see Figures 2 and 3 in the current manuscript) to make it clear for the readers.

Comment 5. Line 153: "coordination number […] was 3.1 Å" This seems to be a mistake.

Response: We corrected this mistake in the revised manuscript.

Comment 6. DFT section: Please describe binding modes.

Response: Thanks for the reviewer's comment. Actually, in Supplementary Information (see **Table S7 and S8**), we have described all possible binding modes and corresponding energies of acetylene on Cu_{13}@Gr and hydrogen on Cu_1@Gr. We also added this point to the main text.

Comment 7. DFT section: It is unclear where H_2 dissossicates. Please clarify.

Response: We thank the reviewer for the comment. For Cu_{13}@Gr, the dissociation process of H_2 on Cu_{13}@Gr (see **Figure R1 (i.e., Figure S15 in the revised SI)**) has an energy barrier of 0.12 eV and is exothermic by 1.05 eV in Gibbs energy. The most stable adsorption site of acetylene is the bottom atom of Cu_{13} cluster which bonds with graphene support. The adsorption energy of acetylene is much stronger as 2.06 eV, which origins from acetylene molecules interacting with three Cu atoms via Cu-C bonds.

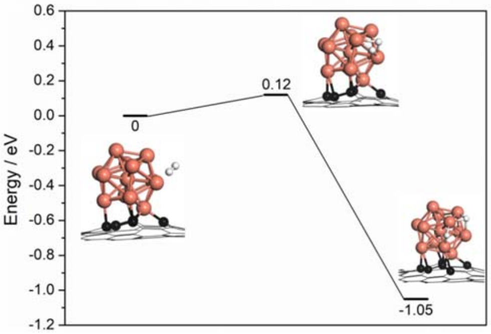

Figure R1. The energy barrier of H_2 activation on Cu_{13}@Gr.

For Cu_1@Gr, the adsorption of acetylene (-1.19 eV) is far stronger than that of H_2 adsorption (-0.29 eV) or H_2 dissociation adsorption (-0.28 eV), indicating the activation of H_2 follows the prioritized adsorption of acetylene on Cu_1/Gr. In other words, acetylene adsorbs firstly at the atomic Cu site via a σ bond with Cu. Then one H_2 gas molecule reacts with the adsorbed acetylene molecule on Cu_1/Gr, which overcomes a barrier of 1.36 eV via a di-σ bond with the atomic Cu site as shown in Figure 4. The following hydrogenation steps are clearly shown in Figure 4, and the bond length of various key intermediates is listed in **Table R1** (we added the result in the revised supplement information, **Table S5**).

Figure R1 (we added the result in the revised supplement information, **Figure S12**) shows the activation transition states (TS1) structure of the first H_2 gas molecule. **Figure R2** (we added the result in the revised supplement information, **Figure S13**) shows the only imaging frequency direction of TS1, which can be found that H-H bond tends to be broken and C-H bond will be formed. The reaction energies, activation energies are provided in SI.

Table R1. The bond length (Å) of key intermediates during acetylene hydrogenation on Cu_1@Gr.

Intermediate	Bond length (Å)				
	C-C(C_2Hx)	C(C_2H_x)-Cu	H(H_2)-Cu	H-H(H_2)	C-H(H_2)
C_2H_2*	1.26	1.97	-	-	-
TS1	1.28	2.10	1.65	1.06	1.44
C_2H_4*	1.40	2.08	-	-	-
TS2	1.44	2.15	1.63	1.22	1.43

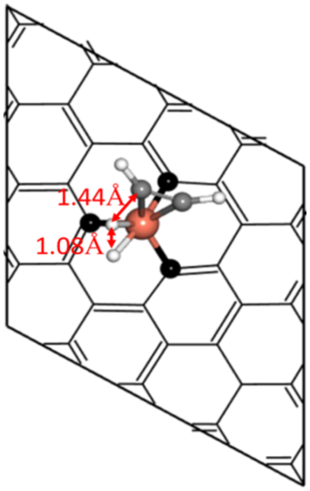

Figure R2. The activation transition states (TS1) of the first H_2 gas molecule on Cu_1@Gr. Color code: Cu (orange), C in graphene (black), C in reactant/intermediates/product (gray) and H (white).

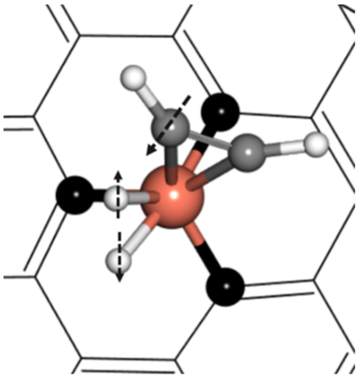

Figure R3. The vibration direction of the only imaging frequency of TS1 on Cu_1@Gr. (Energy of the only imaging frequency is 160 meV).

Reviewer #2
Comments:
Although the manuscript includes structual characterization and computation studies of materials, in my opinion, this work does not represent the requisite conceptual or functional leap for publication in Nature Communications. The obtained materials have not been demonstrated, neither a good reproducibility or to provide a functional benefit, and the performances, claimed but not demonstrated, for what should be the better catalyst Cu_1/ND@G, is in line with a great deal of previous work on nontoxic and inexpensive metals. While the structural study is indeed interesting but not enough, the work seems more suitable for published elsewhere after revisions. Some specific comments follow.

Response: We wholeheartedly thank the reviewers for spotting the mistakes (such content was deleted by accident) in our original submission. A similar point was raised by Reviewers 1 and 3 as well. In this revised version, we added a few paragraphs in the main text to discuss the catalytic activities (see the paragraphs in blue on Page 10, Line 15 to Page 11, Line 18).

We also added the details for the DFT simulations to the Methods section. We also expanded the discussion of the DFT simulation results in the main text (see the paragraph in blue on Page 12, Line 5 and Page 13, Line 2).

Comment 1: The structure feature are defined unique even if literature say the contary.

Response: We thank the reviewer for the comment. Actually, to the best of our knowledge, the atomically dispersed Cu over ND@G as well as small Cu clusters over ND@G were not reported by other groups. Also, we have demonstrated for the first time that Cu species are anchored over the ND@G substrate through the Cu-C binding (Figure 3), which is important for the future design and construction of this type of atomically dispersed or cluster catalyst.

Comment 2: The discriminant factor to prepare Cu_1/ND@G versus Cu_n/ND@G is just temperature at which reduction via H_2 is performed, 200°C or 600°C for 1 hour respectively, My feeling is that synthetic pathway is not well defined and/or reproducible.

Response: We thank the reviewer for the concerns. We carefully checked the reproducibility in synthesis. The procedures for preparing these two catalysts were repeated at least three times in our work, and we confirmed that the synthetic pathway is reproducible.

Comment 3: To discard mixed valence states it could helpful electronic paramagnetic resonance (EPR) measurements.

Response: Special thanks to the reviewer's suggestion for improving our manuscript. According to the reviewer's suggestion, we conducted EPR measurements. The EPR was a sensitive method and only characterized the hyperfine structure of Cu^{2+} species due to unpaired electrons in the outermost layer. But $[Cu\text{-}O\text{-}Cu]^{2+}$, CuO and Cu^{+} showed no EPR activity. As shown in Figure R4, in the spectrum of bare ND@G, a tiny signal can also be detected, which is probably originated from the oxygen radicals on ND@G. The catalysts (Cu_1/ND@G and Cu_n/ND@G) containing 0.25% of Cu showed barely visible fingerprint of Cu^{2+} species, suggesting a relative low content of isolated Cu(II) ions in all investigated samples. This result further confirms that the active sites are not isolated Cu(II) oxide, and agrees well with our XPS and XAFS results.

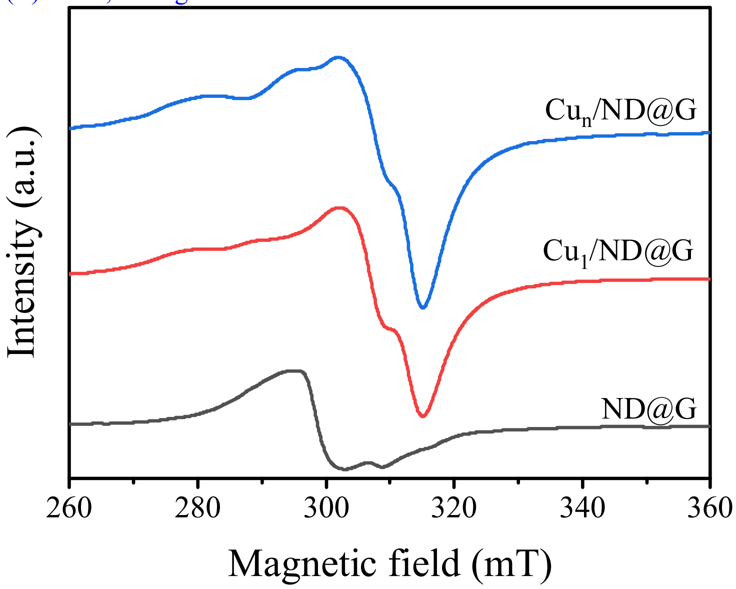

Figure R4 EPR spectra of ND@G support, Cu_1/ND@G and Cu_n/ND@G catalysts recorded at low temperature (77 K).

Comment 4: Authors started saying in intro that relatively high temperature are needed in case of Ceria or Fe oxide while measuring activity at 200 °C, it is demonstrated the contary, in fact Fe(III) does work at 150 °C and with an higher GHSV.

Response: We thank the reviewer for pointing this out for us. We are sorry for our inaccurate statement in the previous introduction, especially about Fe oxide. Indeed, Fe(III) does work at 150°C with an relatively higher GHSV (4000 h^{-1}) than that in our work (3000 h^{-1}). In the design of our work, we studied about Fe oxide catalyzing acetylene hydrogenation (*ACS Catal.* 2017, 7, 3121-3729; *J. Am. Chem. Soc.* 2018, 140, 8827-8832) and got inspired from these works. The work not only introduced Fe(III) single sites (enzymes) into heterocatalysis hydrogenation but also

enabled relevant hydrogenation in industry. So, we have made the correction according to the reviewers' comments, and new references are now added in the revised manuscript.

Comment 5: Even if authors spent 80% of the text trying to define structures, with a very poor report on catalytic activity, no any study on structure before and after the continuous hydrogenation of acetylene in an ethylene flow at working temperature has been reported.
Response: We thank the reviewer for the comment. We added a few paragraphs in the main text (see from Page 10 to Page 11, marked blue font) to discuss the catalytic activity. About the question of studies on catalyst structures before and after continuous acetylene hydrogenation, we have characterized the structure of Cu_1/ND@G after 60 hours test by HADDF-STEM (**Figure S7**) and XAFS (**Figure S8**) and discussed the results in manuscript (see page 11, line 1).

Comment 6: Most, if not all, of the figures related to TEM characterization in the manuscript seems of poor resolution. In several cases this makes deciphering the structural nuance of these materials and the key structural transition difficult. What I mean is thst it could be possible a different percentage of copper clusters but it cannot be discarded in Cu_1/ND@G. Also EXAFS in Fig. 2b shows a shoulder on red line in the region of Cu-Cu distance which may suggest a presence of copper clusters as well.
Response: We thank the reviewer for pointing this out for us. We have improved Figure 1 using clearer images, particularly Figure 1d in the revised manuscript. For light metal like Cu, its contrast with the supports is not so obvious. However, we still got totally different structural information through HADDF-STEM. And we can also confirm its atomic dispersion by the XAFS analysis. Maybe there are tiny amount of Cu clusters on Cu_1/ND@G. However, the absence of Cu-Cu scattering in R-space spectrum of Cu_1/ND@G indicates that atomically dispersed Cu is the dominant Cu species. The shoulder located at 2.5 Å is different from the Cu-Cu coordination peak located at 2.2 Å. The fitting results (as shown in Figure 2e) indicates that this shoulder peak still atrributes to the structure of atomic Cu, i.e. the contribution from 2nd Cu-C shell. Surely it is still open for discussion.

Comment 7: A fastidious "superlative writing" pervades the whole paper where the authors repeatedly use the terms "excellent, unique, outstanding" and so on. Never I could read something like "To the best of our knowledge"
The authors repeatedly state that the material shows "remarkably high selectivity" or exhibits "good stability", without providing any sort of meric that makes this true (Figure 3 is not enough). One could argue the contrary as no comparisions with other inexpensive and relatively nontoxic metals such as Fe, Ti, or Zr, is discussed.
Response: We appreciate the reviewer for his/her comment that inspired us to make a comparison among the existing inexpensive and relatively nontoxic metals. As shown in **Table R2**, compared with previously reported non-noble metal catalysts, our catalyst shows significant advantage (higher metal utilization ratio and selectivity) over the existing catalysts. Compared to Fe(III) catalysts and single atom Ni catalysts, our method is much simpler, as others need expensive precursor (e.g., MOF) or complicated preparation method. More importantly, Cu_1/ND@G reported here shows much higher intrinsic activity (see **Figure R5**) than Ni_3Ga (Adv. Mater. **2016**, *28*, 4747), and Na-Ni@CHA (*J. Am. Chem. Soc.* **2019**, *141*, 9920, this manuscript was online after our submission). However, it is a pity that the reported studies about Fe, Ti or Zr fail to precisely describe the information about TOF (e.g., actual activity values at low conversions). Therefore, we could not provide more comparison information for the Fe, Ti, and Zr catalysts. However, following the suggestion of the reviewer, we have tuned down our claim and removed some of "excellent, unique, outstanding" and added "To the best of our knowledge" in the revised manuscript.

Table R2. Catalytic performance of different non-noble metals based acetylene hydrogenation.

catalyst	Metal loading (%)	T (^oC)	Feed (C_2H_2:H_2:C_2H_4)	Conversion (%)	Selectivity (%)	Ref.
$[Fe^{III}-(H_2O)_6][Fe_2^{III}(\mu-O)_2(H_2O)_6]_{1/2}\{Ni^{II}_4[Cu^{II}_2(Me_3mpba)_2]_3\}\cdot 72H_2O$	2.8	150	1 mL/min (1.2% C_2H_2/C_2H_4); 2 mL/min H_2	100	< 90	*J. Am. Chem. Soc.* **2018**, *140*, 8827
Fe^{III}-nZrO_2	10	150		90	> 85	
nFe_2O_3-nTiO_2	0.5	200		100	90	
$Al_{13}Fe_4$	-	200	0.5%:5%:50%	~95	81-84	*Nature Mater.* **2012**, *11*, 690
Cu_1/ND@G[a]	0.25	200	1%:10%:20%	95	98	This work
Cu_1/ND@G[b]	0.25	180	1%:10%:20%	100	96	
Na-Ni@CHA	3.5	180	0.5%:8%:50%	100	90	*J. Am. Chem. Soc.* **2019**, *141*, 9920.
Ni SAc/N-C	-	200	0.5%:10%:50%	90	90	*Chem. Commun.* **2017**, *53*, 11568.
Ni_3Ga	-	200	0.5%:10%:50%	90	77	*Adv. Mater.* **2016**, *28*, 4747.

a. GHSV = 3000 h^{-1}; b. GHSV = 2400 h^{-1}.

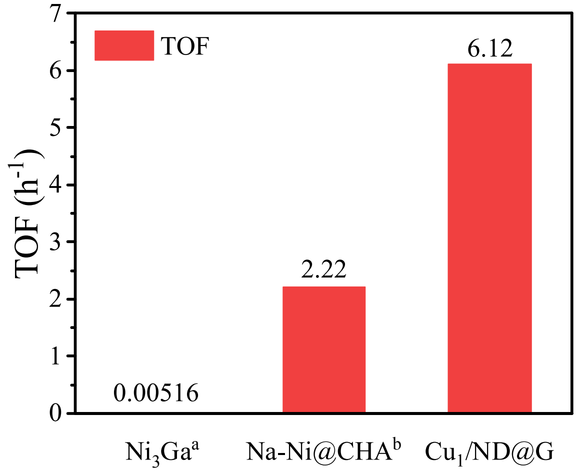

Figure R5. TOF values of non-noble metals for acetylene hydrogenation (T = 80 ^oC; at below 20% acetylene conversion, in the kinetic region; a. *Adv. Mater.* **2016**, *28*, 4747; b. *J. Am. Chem. Soc.* **2019**, *141*, 9920).

Reviwer #3

Comments:

1. Nanodiamonds are of course far of being "cheap and earth-abundant" materials, thus undermining the advantage of using Cu as a catalytic metal instead of, i.e., Pd. Notice that the support constitutes >99 wt.% of the material and that the commercial catalyst is made of simple alumina. Please also notice that solid catalysts with expensive metals are generally recycled after a catalyst lifetime which minimizes losses of the expensive metal. The case here resembles to me that of "earth-abundant" metals with extremely expensive and difficult to prepare ligands in organometallic chemistry. These comments do not underestimate the academic value of the results here presented with Cu_1 and the potential expansion of these results to other more available supports, however, the particularity of the nanodiamond/graphene material for a real application must be accepted.

Response: We appreciate the reviewer for the nice comment. Surely, we need to say that nanodiamonds are not natural "earth-abundant" materials. However, because of the huge price advantage of Cu compared to Pd (i.e., the price of Pd is dozens of times higher than that of Cu),

the nanodiamond/graphene supported Cu catalyst is still far cheaper than the supported Pd catalyst. As the reviewer mentioned, the recycle of supported noble metals minimizes the loss of expensive metals. We also thank the reviewer to point out that "These comments do not underestimate the academic value of the results here". However, we added a sentence to tune down our claim in the revised manuscript (see page 5, paragraph 2).

2. The industrial semi-hydrogenation of acetylene under front-end conditions (those here simulated) operates, at least, at 99.9% conversion and 99% selectivity. In the industrial process, acetylene must be converted from 1 mol% to < 10 parts-per-million, otherwise the subsequent caalyst for polyethylene manufacture is poisoned, and potential explosive acetylide species can be formed. This requisite is not arguably but a "must" for any catalyst for semi-hydrogenation of acetylene. In the other hand, the formation of ethane affects to the process "only" in economic terms, and its value can acceptable, but not the 98% conversion. The reaction must be run in order to get > 99.9% conversion, and then see if the Cu_1 catalyst can still keep a 95% selectivity.
Response: We thank the reviewer for the nice suggestion. Following the suggestion of the reviewer, we have conducted the catalytic test under 100% acetylene conversion and added the result in the revised Supplementary Information (Figure S11). As shown in Figure S6, the Cu_1/ND@G catalyst remains 96% selectivity and shows robust stability at full degree of acetylene conversion.

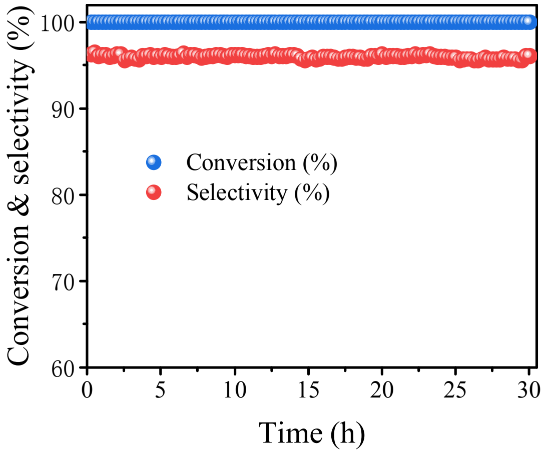

Figure R6. Durability test on Cu_1/ND@G at 180 °C with an 100% acetylene conversion and 96% ethylene selectivity for 30 h. (Reaction condition: 1% C_2H_2, 10% H_2, 20% C_2H_4 gas mix balanced with He; GHSV = 2400 h^{-1}.)

3. The manuscript is in general well-written and well-presented, however, I cannot find the paragraph commenting the catalytic results (Figure 3 and some Figure in the SI). The paragraph may have been skipped during the manuscript production and the reader can only interpret the results from the graphics. Other minor typos: Page 7, line 111 "both catalysts", P13, L209 "catalysts"; P14, L219 "° C", "were measured", L226 "cooled", P15, L243 "calculated".
Response: We wholeheartedly thank the reviewers for spotting the mistakes (such content deleted by accidents) in our original submission. A similar point was raised by Reviewers 1 and 2 as well. In this revised version, we added a few paragraphs in the main text to discuss the catalytic activities (see the paragraphs in blue on Page 10, Line 15 to Page 11, Line 18).

REVIEWERS' COMMENTS:

Reviewer #1 (Remarks to the Author):

The authors improved the text significantly by adapting it to the review comments. The previously raised questions of the review have been properly addressed. In the introduction, the authors provide an appropriate overview of the state of research concerning selective hydrogenation catalysts. The results from catalytic tests are now embedded in a concise frame of text. DFT studies on Cu1-adatoms and Cu13-clusters corroborate the experimental results. I recommend the manuscript for publication, however only, after the following further issues are addressed:

Did the DFT calculations indicate to which extent charge transfer between Cu1/Cu13 to the substrate takes place?

p.13: The authors state that the reason for the high selectivity of the Cu1 catalyst is that the energy of TS2 (towards full hydrogenation) surmounts the energy required to desorb the adsorbate as C_2H_4 (1.27 eV vs 1.08 eV, $\Delta E=0.19$ eV). Strictly following the same argument, however, even semihydrogenation should be unfavorable – the energy required to form TS1 is larger than the energy to desorb acetylene 1.19 eV vs. 1.36 eV, $\Delta E=0.17$ eV. The argumentation here seems contradictive.

p.13: I further recommend to include S14 into the manuscript for a direct comparison of DFT studies on Cu1 and Cu13. A short summary of the most important results from the calculation over the Cu13-cluster would also benefit the text.

p.14: Please state the reaction conditions for the mentioned conversion and selectivity values.

Reviewer #2 (Remarks to the Author):

The revised version of Dr Ma and co-workers dealing with Cu1 species for semi-hydrogenation of acetylene supported on nanodiamond-graphene has been well improved. I have to confess that only in this new form, where important details and a new paragraph about catalysis has been added, I could find the novelty of the work.
I would suggest as minor point to move Tables S3 in main text. I think a comparison with other catalysts employed for acetylene hydrogenation may sound very useful for the Readers of the journal.

Reviewer #3 (Remarks to the Author):

Authors have properly fulfilled most of this Reviewer concerns, however, the 100% conversion reported in Figure S11 must be better quantified, i.e., if it corresponds to >99.0%, >99.9% and so on. Please give the sensitivity of the instrumentation and the exact conversion values, with the error of the measurement, along the time. Only a stable conversion with time higher than >99.95 may have some industrial relevance, since acetylene must be dropped from 1 mol% to, at least, <5 ppm. If so, the manuscript seems publishable in Nat. Commun..

Response to reviewers' comments

Reviewer #1

The author improved the text significantly by adapting it to the review comments. The previously raised questions of the review have been properly addressed. In the introduction, the authors provide an appropriate overview of the state of research concerning selective hydrogenation catalysts. The results from catalytic tests are now embedded in a concise frame of text. DFT studies on Cu_1-adatoms and Cu_{13}-clusters corroborate the experimental results. I recommend the manuscript for publication, however only, after the following further issues are addressed:

(1) Did the DFT calculations indicate to which extent charge transfer between Cu_1/Cu_{13} to the substrate takes place?

Response: Thanks for the reviewer's comment. In previously reported researches, many theoretical works did demonstrate the charge transfer between metals and graphene substrate. For example, the work from Fampiou et al (J. Phys. Chem. C 2012, 116, 6543-6555) indicates that strong binding of Pt13 cluster to point defects of graphene leads to charge transfer (0.48 e) from the cluster to the substrate. Thus, we suspect that charge transfer significantly from Cu_1/Cu_{13} to the single-defect site of graphene, like the case of Fampiou's work, although we didn't perform likewise DFT calculations. What's more, the significant adsorption energy span (-1.26 to -2.06 eV) for acetylene on the possible sites of supported Cu_{13} cluster suggests the electronic effect of substrate on Cu_{13} cluster. The most stable and unstable site of acetylene adsorption is the interface atom that contact with substrates and the top atoms of Cu_{13} cluster respectively, which also prove the electronic effect between graphene substrates to Cu_{13}.

(2) p.13: The authors state that the reason for the high selectivity of the Cu_1 catalyst is that the energy of TS2 (towards full hydrogenation) surmounts the energy required to desorb the adsorbate as C_2H_4 (1.27 eV vs 1.08 eV, E=0.19 eV). Strictly following the same argument, however, even semihydrogenation should be unfavorable – the energy required to form TS1 is larger than the energy to desorb acetylene 1.19 eV vs. 1.36 eV, E=0.17 eV. The argumentation here seems contradictive.

Response: We thank the reviewer for the comment. Beside considering the desorption barrier, the elementary reaction energy should also be considered. The reaction energy from C_2H_2 to C_2H_3 is -0.36 eV, and that from C_2H_4 to C_2H_5 is 0.20 eV, which suggests that the the elementary reaction from C_2H_2 to C_2H_3 is favorable while that from C_2H_4 to C_2H_5 is unfavorable from the view of thermaldynimics. Moreover, as the surface concentration of the C_2H_2* is relatively high, it also could drive the reaction to the forward direction (to a more energy favored intermediates C). In conclusion, the C_2H_4 hydrogeation is unfavorable from both kinetics and thermodynamics.

(3) p.13: I further recommend to include S14 into the manuscript for a direct comparison of DFT studies on Cu_1 and Cu_{13}. A short summary of the most important results from the calculation over the Cu_{13}-cluster would also benefit the text.

Response: We thank the reviewer's suggestion. However, considering the formats requirment and the concise frame of text, we fail to put supplementary figure 11 into main text with regret. But, we do emphasize the comparison of DFT studies on Cu_1 and Cu_{13} in main text (DFTcalculation section) and supplementary figure 16.

(4) p.14: Please state the reaction conditions for the mentioned conversion and selectivity values.

Response: Thanks for the reviwer's comment. Actually, the reaction conditions for the mentioned conversion and selectivity values are stated in the legend of Figure 4.

Reviewer #2

The revised version of Dr Ma and co-workers dealing with Cu_1 species for semi-hydrogenation of acetylene supported on nanodiamond-graphene has been well improved. I have to confess that only in this new form, where important details and a new paragraph about catalysis has been added, I could find the novelty of the work. I would suggest as minor point to move Tables S3 in main text. I think a comparison with other catalysts employed for acetylene hydrogenation may sound very useful for the Readers of the journal.

Response: Special thanks to the reviewer's suggestion. It is a very good suggestion for improving attraction. However, it is a pity that in the current version, four figures including structual and performance characterization are displayed in manuscript. Considering the concise frame and the formats requirment of text, we still decide with regret to placed performance comparision table as supplementary table 3 into supplementary information. But, following the reviewer's advice, we described and emphasized the performance comparisons and mentioned supplementary table 3 in main text (paragraph 8).

Reviewer #3

Authors have properly fulfilled most of this Reviewer concerns, however, the 100% conversion reported in Figure S11 must be better quantified, i.e., if it corresponds to >99.0%, >99.9% and so on. Please give the sensitivity of the instrumentation and the exact conversion values, with the error of the measurement, along the time. Only a stable conversion with time higher than >99.95 may have some industrial relevance, since acetylene must be dropped from 1 mol% to, at least, <5 ppm. If so, the manuscript seems publishable in Nat. Commun..

Response: We thank the reviewer for pointing this out for us. In our work, the reactants and products were analyzed by GC (Agilent 7890A) equipped with a flame ionization detector (FID) and a HP-PLOT AL/S (HP-plot 19091-P-S15, Agilent, 50 m × 0.32 mm × 8 μm) capillary column with He as the carrier gas. The acetylene gas content at about 5 ppm could be effectly detected by our GC-FID detector. So, when the acetylene component couldn't be detected by GC, the content of acetylene is below of 5 ppm. Of course, the 100% conversion reported in supplementary figure 11 is in less quantified, as reviewer's mentioned. Be more strickly speaking, the conversion should be expressed rigorously as higher than 99.95% (> 99.95%). So, we have made correction of supplementary figure 11 according to the reviewer's comments.
